# Differentiating lung neuroendocrine neoplasms from tumor-like infection using CT in patients with ectopic ACTH syndrome

**DOI:** 10.1186/s13244-024-01775-9

**Published:** 2024-08-01

**Authors:** Lan Song, Hui Miao, Zhenchen Zhu, Huijuan Zhu, Jinhua Wang, Xiaoping Xing, Zhaohui Zhu, Yuanyuan Jiang, Ruie Feng, Yu Xiao, Lian Duan, Xin Sui, Qingxing Liu, Linjie Wang, Shi Chen, Wei Song, Zhengyu Jin, Lin Lu

**Affiliations:** 1 Department of Radiology, Peking Union Medical College Hospital, Chinese Academy of Medical Sciences and Peking Union Medical College, Beijing, 100730 China; 2 Department of Neurosurgery, Peking Union Medical College Hospital, Chinese Academy of Medical Science and Peking Union Medical College, Beijing, 100730 China; 3 4+4 Medical Doctor Program, Chinese Academy of Medical Sciences and Peking Union Medical College, Beijing, 100730 China; 4 Department of Endocrinology, Key Laboratory of Endocrinology of National Health Commission, Peking Union Medical College Hospital, Chinese Academy of Medical Sciences and Peking Union Medical College, Beijing, 100730 China; 5 Department of Nuclear Medicine, State Key Laboratory of Complex Severe and Rare Diseases, Peking Union Medical College Hospital, Chinese Academy of Medical Sciences and Peking Union Medical College, Beijing, 100730 China; 6 Department of Pathology, Peking Union Medical College Hospital, Chinese Academy of Medical Sciences and Peking Union Medical College, Beijing, 100730 China

**Keywords:** Ectopic ACTH syndrome, Neuroendocrine tumors, Pulmonary infection, Tomography (x-ray computed)

## Abstract

**Objectives:**

Pulmonary neuroendocrine neoplasms (NENs) are the most frequent cause of ectopic adrenocorticotropic hormone syndrome (EAS); lung infection is common in EAS. An imaging finding of infection in EAS patients can mimic NENs. This retrospective study investigated EAS-associated pulmonary imaging indicators.

**Methods:**

Forty-five pulmonary NENs and 27 tumor-like infections from 59 EAS patients (45 NEN and 14 infection patients) were included. Clinical manifestations, CT features, ^18^F-FDG, or ^68^Ga-DOTATATE-PET/CT images and pathological results were collected.

**Results:**

High-sensitivity C-reactive protein (*p* < 0.001) and expectoration occurrence (*p* = 0.04) were higher, and finger oxygen saturation (*p* = 0.01) was lower in the infection group than the NENs group. Higher-grade NENs were underrepresented in our cohort. Pulmonary NENs were solitary primary tumors, 80% of which were peripheral tumors. Overlying vessel sign and airway involvement were more frequent in the NENs group (*p* < 0.001). Multifocal (*p* = 0.001) and peripheral (*p* = 0.02) lesions, cavity (*p* < 0.001), spiculation (*p* = 0.01), pleural retraction (*p* < 0.001), connection to pulmonary veins (*p* = 0.02), and distal atelectasis or inflammatory exudation (*p* = 0.001) were more frequent in the infection group. The median CT value increment between the non-contrast and arterial phases was significantly higher in NENs lesions (*p* < 0.001). Receiver operating characteristic curve analysis indicated a moderate predictive ability at 48.3 HU of delta CT value (sensitivity, 95.0%; specificity, 54.1%).

**Conclusion:**

Chest CT scans are valuable for localizing and characterizing pulmonary lesions in rare EAS, thereby enabling prompt differential diagnosis and treatment.

**Critical relevance statement:**

Thin-slice CT images are valuable for the localization and identification of pulmonary ectopic adrenocorticotropic hormone syndrome lesions, leading to prompt differential diagnosis and effective treatment.

**Key Points:**

- Lung tumor-like infections can mimic neuroendocrine neoplasms (NENs) in ectopic adrenocorticotropic hormone syndrome (EAS) patients.
- NENs are solitary lesions, whereas infections are multiple peripheral pseudotumors each with identifying imaging findings.
- Typical CT signs aid in localization and creating an appropriate differential diagnosis.

**Graphical Abstract:**

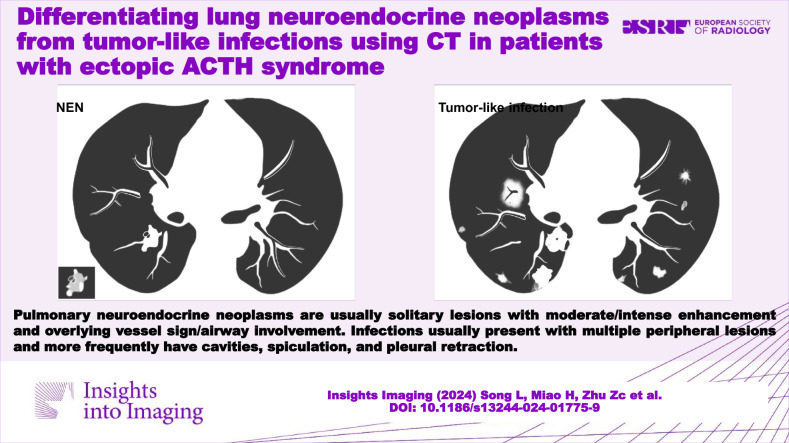

## Introduction

Ectopic adrenocorticotropic hormone (ACTH) syndrome (EAS) is a rare condition with an annual incidence of 0.8 per million [1]. EAS accounts for 9–18% of ACTH-dependent Cushing’s syndrome (CS) and 5–10% of all CS cases [2, 3]. EAS is often associated with rapidly progressive clinical features such as severe hypokalaemia and high ACTH and cortisol levels [4]. Neuroendocrine neoplasms (NENs) are a rare and heterogeneous group of tumors, encompassing well-differentiated neuroendocrine tumors (NETs) and poorly differentiated neuroendocrine carcinomas (NECs) [5, 6]. The common causes of ectopic ACTH production include NETs of the lungs and thymus, pancreatic NETs, pheochromocytomas, and medullary thyroid carcinomas [7]. Lung NETs (LNETs) are the most frequent cause of EAS. Small cell lung carcinoma (SCLC) is another possible etiology of EAS [8]. Timely detection and resection of NETs are essential for good clinical outcomes.

The prevalence of systemic opportunistic infections due to hypercortisolism is higher (up to 51%) in EAS patients compared to patients with Cushing’s disease (21%) [9, 10]. Infection most frequently involves the lungs [11, 12] and is the leading cause of mortality within 90 days of CS treatment initiation, accounting for 31% of deaths and outpacing cardiovascular and cerebrovascular events [13]. *Pneumocystis jirovecii* and species from the genera *Aspergillus*, *Cryptococcus*, and *Nocardia* are well-known pathogens in EAS patients [14–16]. In some cases, pulmonary infections can appear as nodules or masses and mimic lung tumors, leading to unnecessary surgical treatment [12, 17].

Therefore, discriminating LNETs from tumor-mimicking infection lesions is crucial. Diagnosis of EAS usually necessitates a combination of functional imaging methods such as somatostatin receptor scintigraphy, ^18^F-fluorodeoxyglucose (FDG) or ^68^Ga-DOTATATE PET/CT, dynamic observation, and experienced radiologists. Computed tomography (CT) is a widely used imaging method with high sensitivity for identifying pathologically confirmed EAS tumors (85.4%), especially those located in the chest cavity (91.2%) [4]. Additionally, thin-slice CT combined with three-dimensional (3D) postprocessing techniques can show the anatomical relationship between LNETs and airways more clearly. However, some LNETs are very small, occult, or lack specific CT imaging features.

Due to its rarity, the CT features of pulmonary NENs causing EAS have not been systematically elucidated in a large patient population. Therefore, this retrospective study comprehensively evaluated the clinical and imaging features of 59 confirmed EAS cases, involving one group of patients with lung NENs and another group of patients with pulmonary infections. This study also identified CT features that may aid in distinguishing lung NENs from tumor-like pulmonary infections for better clinical management of EAS.

## Methods

### Patient selection

This retrospective study involved 59 EAS patients admitted to our hospital from March 2010 to March 2022. A computerized search of EAS was conducted using the keywords “Ectopic Cushing’s Syndrome”, “Ectopic Adrenocorticotropic Hormone Syndrome”, and “Ectopic Corticotropin Releasing Hormone Syndrome” in the hospital information system. Then, the clinical records and radiological images were carefully reviewed and validated by a senior endocrinologist (L.L., with 25 years of experience in endocrinology) and a senior chest radiologist (L.S., with 18 years of experience in pulmonary imaging diagnosis). This study was approved by the Institutional Review Board of our hospital (I-23PJ175), and written informed consent was waived.

The diagnosis of EAS was based on biological, radiological, and pathological results (Fig. S1). The inclusion and exclusion criteria are listed in the Supplementary Appendix 1.1.

**Fig. 1:**
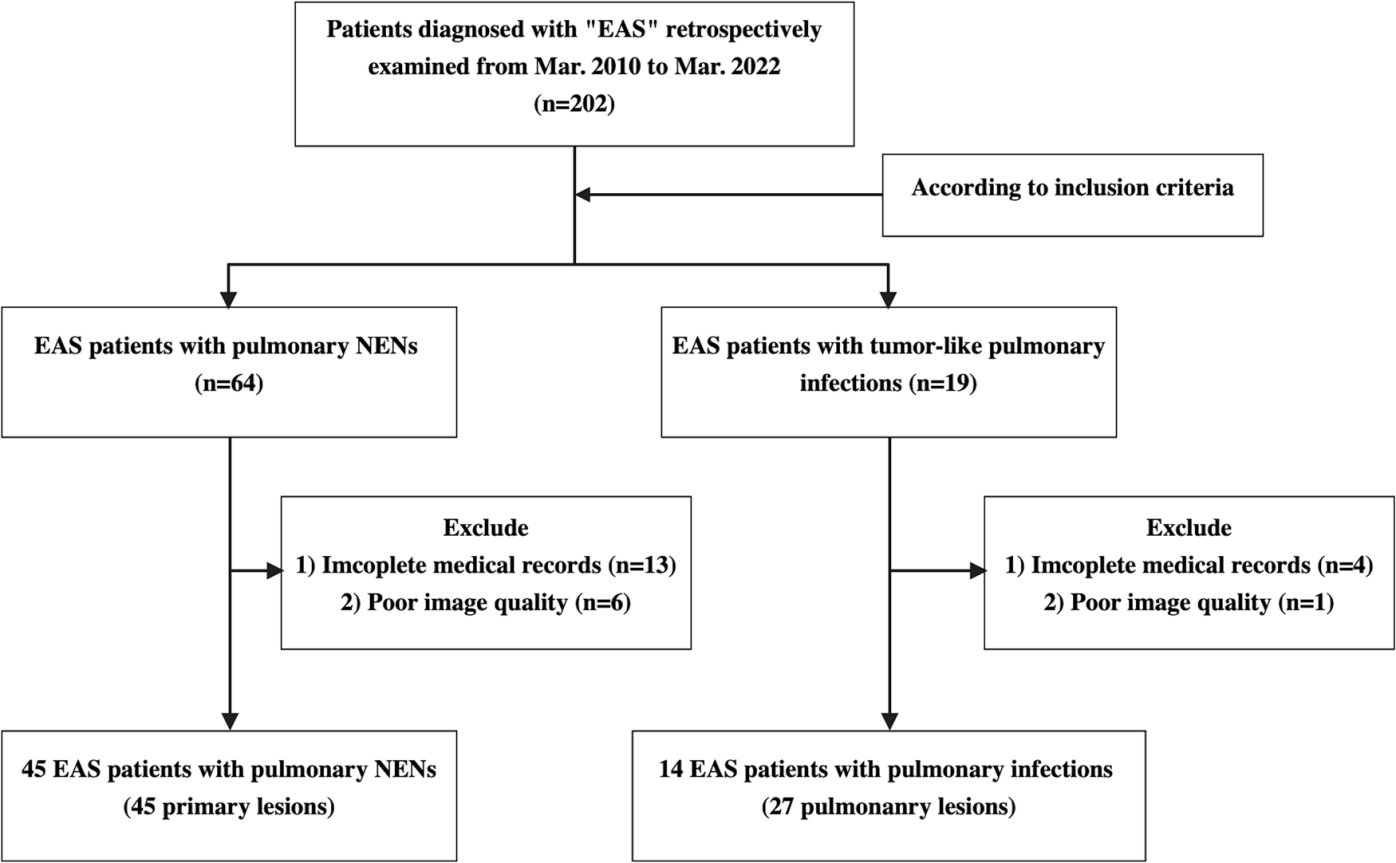
Flowchart of patient eligibility with inclusion and exclusion criteria. EAS Ectopic adrenocorticotropic hormone syndrome, NEN Neuroendocrine neoplasm

Based on the inclusion and exclusion criteria, 45 EAS patients with pulmonary NENs (45 lesions) and 14 EAS patients with pulmonary infections (27 lesions) were included in this study (Fig. 1).

### Clinical information collection

The baseline clinical data from before treatment were obtained from electronic medical records (Table E1). We retrospectively collected clinical features, biochemical indicators of hypercortisolism (serum ACTH, plasma, and urinary cortisol), infection indicators (such as routine blood tests, high-sensitivity C-reactive protein [hs-CRP], and aetiological examination), and pathological results. The details of the endocrine tests are presented in Supplementary Appendix 1.2.

### CT image acquisition and evaluation

The latest CT scans of each patient before surgery or etiology confirmation were used for evaluation. All chest CT examinations were performed from the lung apex to the lung base using various sets of multidetector CT scanners. In addition to non-contrast CT (NCCT), 37 (82.2%) pulmonary NEN patients and nine (64.3%) infection patients also underwent contrast-enhanced CT (CECT). The CECT scans were acquired at 35 s after the injection of 80–100 mL of nonionic contrast material (Ultravist 300, Bayer Schering Pharma AG, Germany) intravenously at a rate of 2.5 mL/s. The details of the scanning parameters and reconstruction methods are provided in Table E2.

CT images of the pulmonary lesions were evaluated according to the published literature [18, 19]. Each CT scan was reviewed on thin-slice CT images (slice thickness ≤ 1 mm) with mediastinal (width: 450 HU, level: 50 HU) and lung (width: 1200 HU, level: −600 HU) window settings using a PACS (GE Healthcare, Chicago, USA). Evaluation was carried out by two senior chest radiologists (L.S. and W.S.) with 18 and 29 years of experience in pulmonary imaging diagnosis, respectively, who were blinded to pathological and pathogenetic testing results. The two readings were performed on the same patient independently on the same day, and differences were resolved through consensus. CECT images were also evaluated if available. The CT morphological features of each lesion and adjacent pulmonary abnormalities were assessed. The involvement of the airway was also evaluated using coronal, sagittal, three-dimensional, and minimum intensity projection (MinIP) reconstruction images, when necessary. A detailed introduction and definitions of the CT features are described in the Supplementary Appendix 1.3 and Table E1. The changes in lung lesions were evaluated when serial chest CT scans were available. The quantitative measurements from the two radiologists were averaged, and the consensus on each qualitative evaluation was documented.

### Functional imaging results

The results of somatostatin receptor scintigraphy, ^18^F-FDG or ^68^Ga-DOTATATE-PET/CT were collected if available and were confirmed by an experienced nuclear medicine physician (Z.Z., with 28 years of experience in nuclear medicine imaging diagnosis). The detailed protocols are described in Supplementary Appendix 1.4–1.6.

### Statistical analysis

Data were expressed as the mean ± standard deviation (SD) or median (Q25, Q75). Chi-square or Fisher’s exact tests, Student’s *t*-test, Mann–Whitney *U* tests and one-way ANOVA were used when appropriate. Cut-off values, areas under the curve (AUCs) and sensitivity and specificity values were obtained by receiver operating characteristic (ROC) analysis. All statistical analyses were performed using SPSS 26.0 (SPSS Inc., Chicago, IL, USA). *p* < 0.05 was considered statistically significant.

## Results

### Patient demographics and clinical features

This study included 72 pulmonary lesions from 59 EAS patients. Clinical features and laboratory results are summarized in Tables 1, 2 and Table E3. Forty-five EAS patients with confirmed pulmonary NENs were diagnosed at baseline (28/45, 62.2%) or during follow-up (17/45, 37.8%) with solitary primary NENs, comprising 32 G1 NETs (71.1%), nine G2 NETs (20.0%) and four SCLCs (8.9%). Among 14 pulmonary infection patients with EAS, only two thymus NENs were found to cause EAS, while the other 12 (85.7%) retained a diagnosis of occult EAS even after follow-up. Lower finger oxygen saturation (95.0% vs. 98.0%, *p* = 0.01) and higher occurrence of expectoration (21.4% vs. 2.3%, *p* = 0.04) were observed in patients with infection compared to those with NENs (Table 1). The hs-CRP was 17.8-fold higher in the infection group than in the NEN group (14.2 mg/L vs. 0.8 mg/L, *p* < 0.001; Table E3). Additionally, most clinical manifestations and biochemical EAS results such as serum cortisol, ACTH, and 24-hour urinary free cortisol (UFC) levels did not significantly differ between the two groups (Tables 1 and 2, all *p* > 0.05).

**Table 1:** Patient demographics and clinical features between EAS with pulmonary NENs and infection

	EAS with pulmonary NENs (*n* = 45)	EAS with infection (*n* = 14)	*p* value
Gender			1.00
Male	24 (53.3%)	7 (50%)	
Female	21 (46.7%)	7 (50%)	
Age (years)	41.8 ± 17.3	48.9 ± 18.5	0.19
Duration of symptoms (months)	20.0 (11.8, 34.5)	17.5 (3.5, 99.0)	0.97
Time from symptoms onset to diagnosis	14.0 (6.0, 24.0)	8.0 (3.0, 35.5)	0.57
Time of follow-up (months)	17.5 (6.3, 40.0)	30.5 (5.5, 53.0)	0.56
Clinical features			
Hypertension	36/43 (83.7%)	12/14 (85.7%)	1.00
Diabetes mellitus/ glucose intolerance	31/39 (79.5%)	10/14 (71.4%)	0.71
Osteoporosis	23/25 (92.0%)	11/14 (78.6%)	0.33
Hypercortisolism	37/45 (82.2%)	10/14 (71.4%)	0.45
Hypopotassemia	39/44 (88.6%)	12/13 (92.3%)	1.00
Smoking history (Yes)	15/45 (33.3%)	7/14 (50.0%)	0.35
Adrenalectomy before diagnosis	6/44 (13.6%)	5/14 (35.7%)	0.11
Fever	1/44 (2.3%)	2/14 (14.3%)	0.14
Cough	3/44 (6.8%)	3/14 (21.4%)	0.15
Expectoration	1/44 (2.3%)	3/14 (21.4%)	**0.04**
BMI (kg/m^2^)	25.7 ± 3.3	26.8 ± 4.7	0.35
Finger oxygen saturation (%)	98.0 (96.0, 98.0)	95.0 (95.0, 97.0)	**0.01**
Clinical outcome (%)			0.09
Death	0	2 (22.2%)	
Survival	20 (100.0%)	7 (77.8%)	
Pathology or pathogen			
NET G1	32 (71.1%)	-	
NET G2	9 (20.0%)	-	
SCLC	4 (8.9%)	-	
Cryptococcus	-	4 (26.7%)	
Nocardiosis	-	2 (13.3%)	
Aspergillus	-	1 (6.7%)	
Other*	-	7 (48.1%)	

* 3 cases with granulomatous inflammation and 4 cases with other fungi

*BMI* body mass index

**Table 2:** Laboratory, HDDST, and functional imaging tests of EAS diagnosis

	EAS with pulmonary NENs (*n* = 45)	EAS with infection (*n* = 14)	*p* value
Number of lesions	45	27	
Laboratory tests of EAS			
Serum potassium (mmol/L) (normal Range: 3.5–5.5 mmol/L)	2.7 ± 0.8	2.5 ± 0.6	0.24
Preoperative ACTH (pg/mL) (normal Range: < 46 pg/mL	157.0 (99.6, 215.0)	289.0 (114.0, 318.0)	0.22
Morning serum cortisol (μg/dL) (normal range: 4–22 μg/dL)	41.6 (31.1, 60.8)	50.9 (31.8, 61.8)	0.89
24-h UFC (μg/24 h) (normal range: 12–103.5 μg)	1483.8 (839.2, 1860.0)	1643.1 (935.6, 3917.5)	0.36
HDDST unsuppressed patients (%)	22/40 (55.0%)	11/13 (84.6%)	0.10
BIPSS no central-to-peripheral gradient patients (%)	34/34 (100%)	7/8 (87.5%)	0.19
SRS (Octreotide scan) positive lesions	7/33 (21.2%)	2/25 (8%)	0.28
^ 18^F-FDG PET/CT positive lesions	28/40 (70.0%)	20/26 (76.9%)	0.59
PET-CT SUV_max_	1.3 (0.9, 3.1)	2.4 (1.2, 5.7)	0.07
^ 68^Ga-DOTATATE PET/CT positive lesions	6/13 (46.2%)	1/13 (7.7%)	0.07

*24-h UFC* 24-h urinary free cortisol, *HDDST* high-dose dexamethasone suppression test, *BIPSS* bilateral inferior petrosal sinus sampling, *CT* computed tomography, *SRS* somatostatin receptor scintigraphy, ^1*8*^*F-FDG PET/CT* ^18^F-Fluorodeoxyglucose positron emission tomography/computed tomography, ^*68*^*Ga-DOTATATE PET/CT* ^*68*^*Ga-1,4,7*10-tetraazacyclododecane-1,4,7,10-tetraacetic acid-D-phenylalanine 1-tyrosine 3-threonine 8-octreotide peptide positron emission tomography/computed tomography

### CT imaging features

**Fig. 2:**
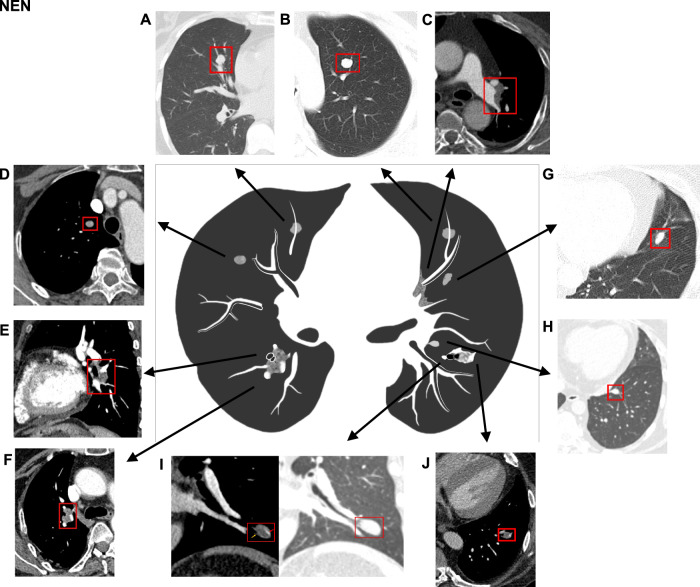
Representative CT images and schematics of pulmonary abnormalities of pulmonary neuroendocrine neoplasms in patients with ectopic ACTH syndrome. **A** Solitary solid nodule in the right middle lobe. **B** Solitary solid nodule in the left upper lobe. **C** Central lesion adjacent to the left hilar region. **D** Solid nodule with moderate enhancement. **E** Solid nodule related closely to adjacent broncho-vascular bundles. **F** Solid nodule with overlying vessel sign. **G** Solid nodule with a clear boundary, no cavity, spiculation, and pleural retraction sign. **H** Occult micro-nodule mimic a cross-section of blood vessels. **I** Solid nodule with subsegmental bronchi involved (yellow arrow) and overlying vessel sign (red arrow). **J** Solid nodule with subsegmental bronchi involved

**Fig. 3:**
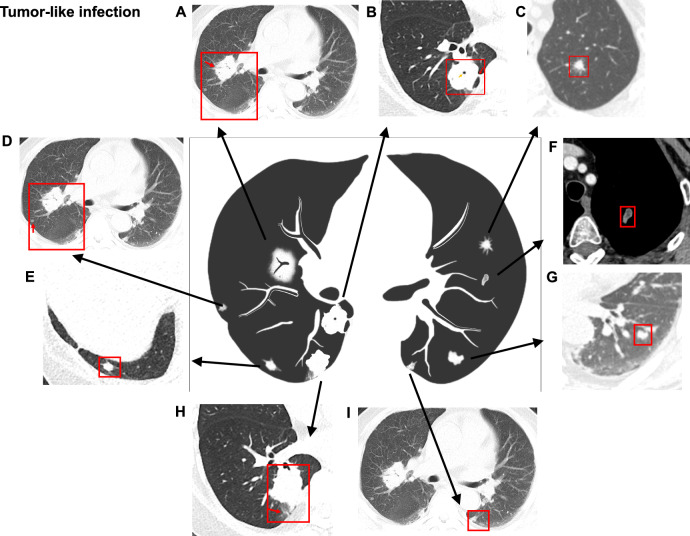
Representative CT images and schematics of pulmonary abnormalities of tumor-like pulmonary infections in patients with ectopic ACTH syndrome. **A** Tumor-like mass with air bronchogram. **A, D, I** Bilateral multiple lesions in the same patient. **B** Tumor-like mass with accompanying cavity (yellow arrow) and pleural retraction sign (red arrow). **C** Solid nodule with spiculation. **E** Solitary subpleural solid nodule. **F** Solid nodule with mild heterogeneous enhancement. **G** Solid nodule not closely related to the bronchovascular bundle. **H** Tumor-like mass and accompanying distal inflammatory exudation (red arrow)

**Fig. 4:**
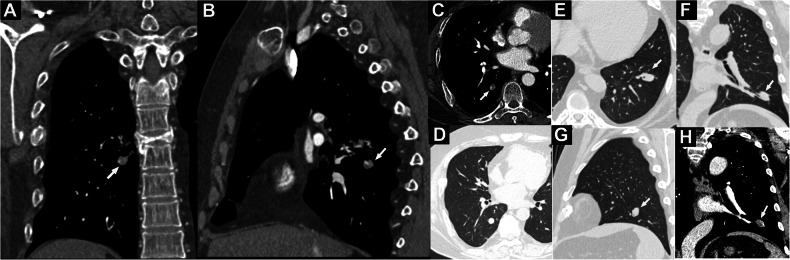
Representative cases of overlying vessel sign and airway involvement. **A–D** A 29-year-old man with a typical carcinoid tumor and associated ectopic Cushing’s syndrome. **A** Coronal, (**B**) sagittal, and (**C**) axial contrast-enhanced CT showing a peripheral solid nodule in the right lower lobe (RLL) with overlying vessel sign (arrow). **D** Axial lung window CT showing a solid nodule with a clear boundary in the RLL. **E**–**H** A 21-year-old man with a typical carcinoid tumor and associated ectopic Cushing’s syndrome. **E** Axial, (**F**) coronal, and (**G**) sagittal 1 mm thin-slice CT at the lung window showing a peripheral solid nodule in the left lower lobe (LLL) and obstructed adjacent subsegmental bronchus (arrow). **H** Contrast-enhanced coronal CT at the mediastinal window showing a solid nodule in the LLL with a clear boundary, inhomogeneous enhancement, and overlying vessel sign (arrow)

**Fig. 5:**
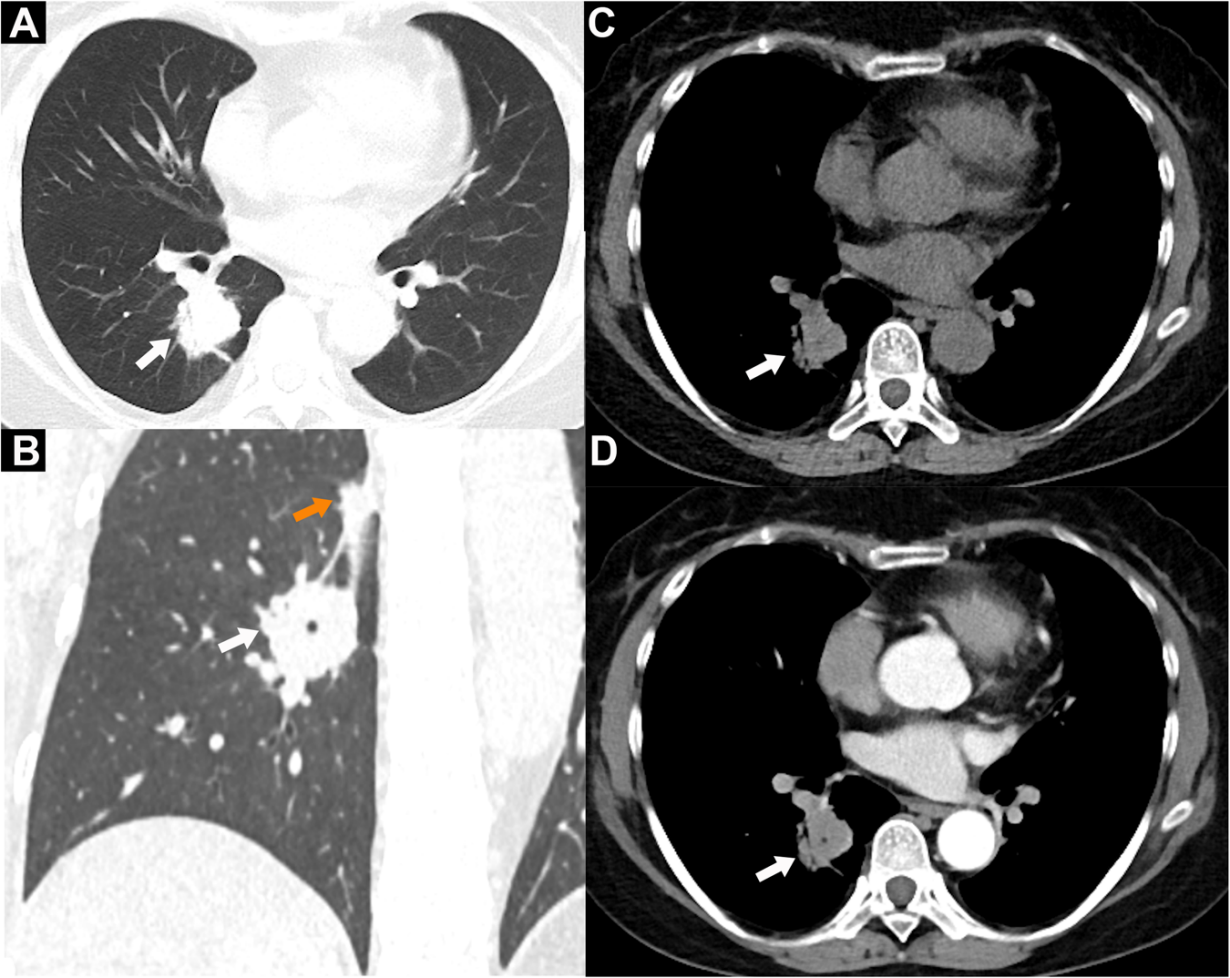
Tumor-like pulmonary infection in a 34-year-old woman with confirmed ectopic Cushing’s syndrome (CS) (**A**–**D)**. Whole body ^18^F-FDG PET/CT scan showing two fluorodeoxyglucose-avid pulmonary lesions in the right lower lobe (RLL) with a maximal standard uptake (SUV_max_) up to 12.4 (big lesion) and 5.7 (small lesion) and no other abnormalities. Technetium-99^m^ somatostatin receptor scintigraphy also showed slightly increased uptake in the pulmonary mass and nodule in the RLL. **A** Axial and (**B**) coronal non-contrast chest CT at the lung window showing a mass (arrows, diameter 3.1 cm) in the RLL and a nodule (orange arrow in **B**, diameter 1.1 cm) beside the right oblique fissure. **A, C, D** An air bronchogram, lobulation, and spiculation were present in the mass. **C** Non-contrast and (**D**) contrast-enhanced axial CT at the mediastinal window showing homogeneous weak enhancement of about 15 HU increments (arrows). **B, D** A thick-wall cavity in the mass and (**A**) pleural retraction is visible. The two lesions were surgically removed and misdiagnosed as ACTH-secreting lung neuroendocrine tumors. Histopathological results confirmed pulmonary cryptococcosis with ACTH negativity, based on immunohistochemistry. The CS symptoms and laboratory tests were not resolved following surgery

The CT imaging features of NENs and infections are summarized in Tables 3 and 4. The median maximal long-axis were 13.0 mm in the infection group, and 10.5 mm in the NEN group (*p* = 0.59). Overlying vessel sign and airway involvement were more frequent in the NEN group than in the infection group (66.7% vs. 18.5%, 88.9% vs. 22.2%, both *p* < 0.001) (Figs. 2–4; Table 3; Fig. E2; Video E1). However, cavity (55.6% vs. 0, *p* < 0.001), spiculation (22.2% vs. 2.2%, *p* = 0.01), and pleural retraction sign (48.1% vs. 6.7%, *p* < 0.001) were more common in the infection group (Figs. 3 and 5, Table E4 and E5). NENs were more likely to connect to pulmonary arteries (18/45, 40.0%), whereas infections were more likely to connect to pulmonary veins (16/27, 59.3%, *p* = 0.02). Among 57 lesions (37 NENs and 20 infections) receiving both NCCT and CECT scans, the median increased enhancement value (i.e. delta CT value) in the NEN group was significantly higher than in the infection group (50.7 HU vs. 19.1 HU, *p* < 0.001) (Figs. 2–5, Fig. E3), with 13 (35.1%) NEN patients and zero infection patients exhibiting an enhancement greater than 60 HU (*p* = 0.001). ROC curve analysis showed that the delta CT value had a moderate predictive ability of 48.3 HU in distinguishing pulmonary NENs from infections (AUC, 0.774; 95%CI, 0.655–0.894; *p* < 0.001; sensitivity, 95.0%; specificity, 54.1%).

**Table 3:** Patient-level morphological features and lesion location on chest CT between EAS with pulmonary NENs and infection

	EAS with pulmonary NENs (*n* = 45)	EAS with infection (*n* = 14)	*p* value
Number of pulmonary nodules/masses per patient*			**0.001**
Single	40 (88.9%)	7 (50.0%)	
2–3	4 (8.9%)	2 (14.3%)	
> 3	1 (2.2%)	5 (35.7%)	
Accompanied clustered nodules	0 (0%)	1 (7.1%)	0.24
Pleural effusion	9 (20.0%)	5 (35.7%)	0.29
Mediastinal or hilar lymph node enlargement	6 (13.3%)	4 (28.6%)	0.23
Lobar location of pulmonary lesions per patient			**0.002**
Left upper lobe	11/45 (24.4%)	0	
Left lower lobe	8/45 (17.8%)	2/14 (14.3%)	
Right upper lobe	5/45 (11.1%)	2/14 (14.3%)	
Right middle lobe	12/45 (26.7%)	3/14 (21.4%)	
Right lower lobe	8/45 (17.8%)	1/14 (7.1%)	
Bilateral multiple lesions	0	4/14 (28.6%)	
Mixed (multiple lobes involvement of right lung)	1/45 (2.2%)	2/14 (14.3%)	
Total number of lesions	45	27	
Lesion site			0.62
Left lung	19 (42.2%)	9 (33.3%)	
Right lung	26 (57.8%)	18 (66.7%)	
Lobar site of each lesion^#^			0.70
Upper/middle lobes	29 (63.0%)	19 (70.4%)	
Lower lobe	17 (37.0%)	8 (29.6%)	
Location of pulmonary lesions			**0.02**
Peripheral	36 (80.0%)	27 (100.0%)	
Central	9 (20.0%)	0	

* The lesions, including primary tumors, intrapulmonary metastases, and intrapulmonary lymph node

^#^ The NEN group has 46 tumors with a case involved multiple lobes

**Table 4:** Lesion-level CT imaging features between EAS with pulmonary NENs and infection

	EAS with pulmonary NENs (*n* = 45)	EAS with infection (*n* = 27)	*p* value
Mean CT attenuation on noncontrast CT (HU)	35.1 (24.6, 41.3)	35.0 (21.5, 48.8)	0.65
Density on non-contrast CT			0.14
Part-solid	0	2 (7.4%)	
Solid	45 (100%)	25 (92.6%)	
Delta CT value (HU)^a^	50.7 (28.0, 67.6)	19.1 (10.1, 37.8)	**< 0.001**
Degree of CT enhancement (HU)			**0.001**
Slight (increment < 30 HU)	10 (27.1%)	13 (65.0%)	
Moderate (30 ≤ increment ≤ 60 HU)	14 (37.8%)	7 (35.0%)	
Intense (increment > 60 HU)	13 (35.1%)	0	
Distance from pleura (mm)	3.1 (0, 11.9)	5.4 (0.5, 9.9)	0.44
Maximum long-axis diameter (mm)	10.5 (8.9, 16.0)	13.0 (8.4, 20.7)	0.59
Lesion type			0.28
Nodule (≤ 3 cm)	41 (91.1%)	22 (81.5%)	
Mass (> 3 cm)	4 (8.9%)	5 (18.5%)	
Shape			0.08
Round or oval	33 (73.3%)	14 (51.9%)	
Polygonal or irregular	12 (26.7%)	13 (48.1%)	
Contour (Smooth)	45/45 (100.0%)	26/27 (96.3%)	0.38
Cavity	0	15 (55.6%)	**< 0.001**
Lobulation			0.14
None	28 (62.2%)	14 (51.9%)	
Shallow	8 (17.8%)	2 (7.4%)	
Deep	9 (20.0%)	11 (40.7%)	
Spiculation	1/45 (2.2%)	6/27 (22.2%)	**0.01**
Pleural retraction sign	3/45 (6.7%)	13/27 (48.1%)	**< 0.001**
Overlying vessel sign	30/45 (66.7%)	5/27 (18.5%)	**< 0.001**
Vascular connections			**0.02**
No discernable vascular connection	11 (24.4%)	9 (33.3%)	
Connected to pulmonary veins only	16 (35.6%)	16 (59.3%)	
Connected to pulmonary arteries only	14 (31.1%)	2 (7.4%)	
Connected to pulmonary artery and pulmonary vein	4 (8.9%)	0	
Airway involvement			**< 0.001**
Main or lobar bronchi involved	11 (24.4%)	2 (7.4%)	
Segmental or subsegmental bronchi involved	29 (64.5%)	4 (14.8%)	
No discernable airway involvement	5 (11.1%)	21 (77.8%)	
Air bronchograms	0	3 (11.1%)	**0.047**
Adjacent pulmonary abnormality			**0.001**
No discernable abnormality	30 (66.7%)	6 (22.2%)	
Accompanied by other nodules	1 (2.2%)^**b**^	1 (3.7%)	
Hyperlucency/emphysema with or without bronchiectasis	2 (4.4%)	3 (11.1%)	
Distal atelectasis or inflammatory exudation	12 (26.7%)	17 (63.0%)	
Lesion status			**< 0.001**
Stable	8 (17.8%)	2 (7.4%)	
Dynamic changing	5 (11.1%)	21 (77.8%)	
Unavailable	32 (71.1%)	4 (14.8%)	

^a^ Delta CT value: CT value increment after contrast enhancement

^b^ An intrapulmonary lymph node was observed intraoperatively and on CT (with pathology confirmed)

**Fig. 6:**
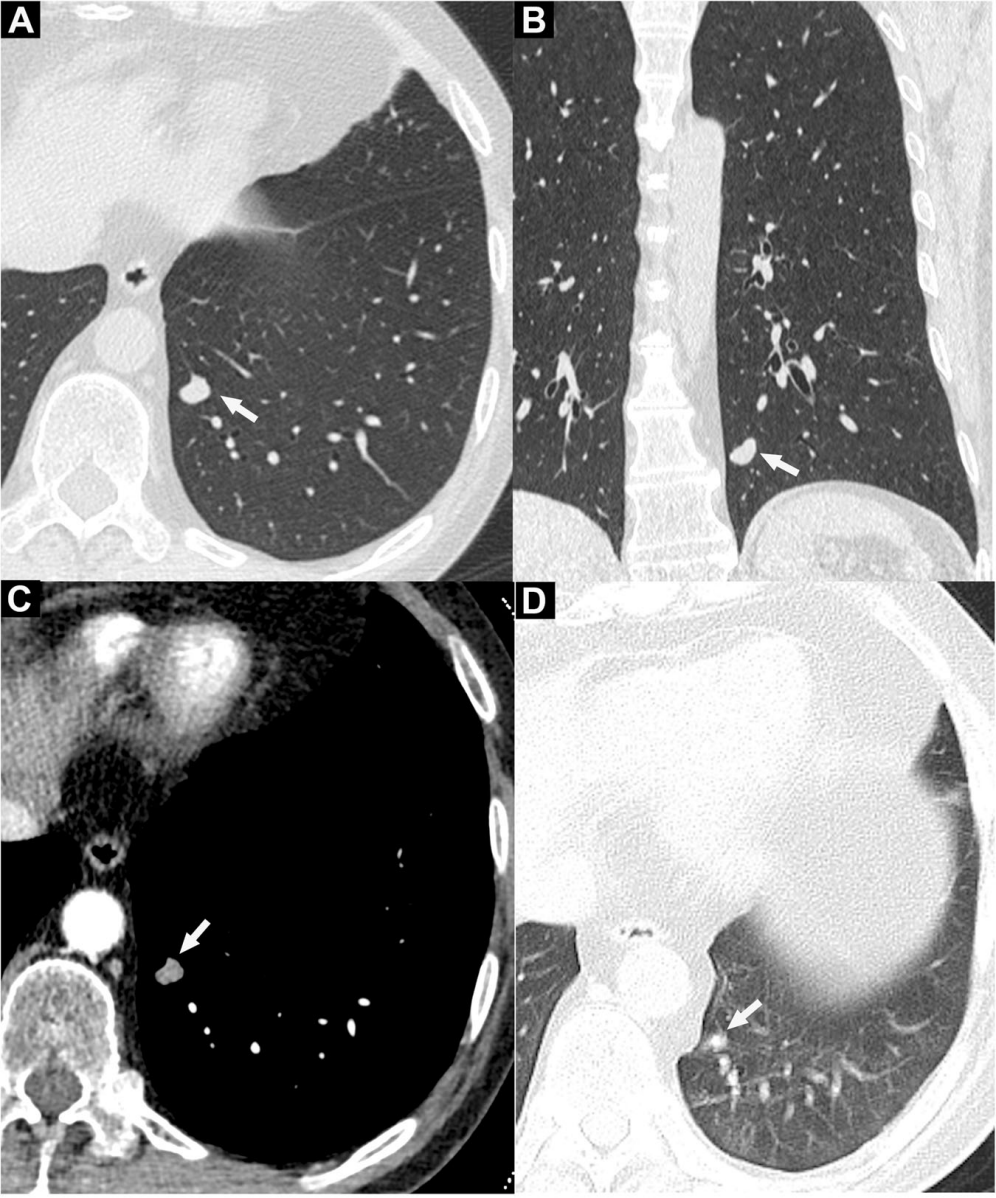
Follow-up CT scans to locate a possible ACTH-secreting tumor in a 48-year-old man with ectopic Cushing’s syndrome (**A**–**D**), The lung nodule was pathologically confirmed as typical carcinoid. No abnormality was found in the somatostatin receptor scintigraphy. Both ^18^F-FDG PET/CT and ^68^Ga-DOTATATE PET/CT did not show increased uptake in the small nodule in the left lower lobe (LLL). Chest CT showing a small peripheral solid nodule (diameter 0.9 cm) in the LLL (arrows in **A**–**C**). **A** Axial and (**B**) coronal 1 mm thin-slice non-contrast CT images at the lung window showing a nodule with a clear boundary and lobulated shape. **C** Thin-slice axial contrast-enhanced CT at the mediastinal window showing moderate homogeneous enhancement (arrow, Δ CT value = 54 HU after contrast injection). **D** The micro solid nodule (diameter 0.4 cm) in the LLL (arrow) was misdiagnosed as a cross-section of a blood vessel on the baseline CT five years prior. The misdiagnosis may have been caused by hindsight bias, poor image quality influenced by a slight respiratory motion artifact, and the 5 mm thick-slice thickness

As shown in Table 3, E5 and Fig. E4, pulmonary NENs were distributed slightly more in the right lung (26/45, 57.8%) than in the left lung (19/45, 42.2%). The NENs were all solitary primary tumors presenting as solid nodules or masses, with one central type of SCLC involving three lobes, two SCLCs, one G2 NET with intrapulmonary metastases, and two G1 NETs accompanied by an invasive adenocarcinoma and an intrapulmonary lymph node in the same lobe (Fig. E5). In the infection group, multiple lesions were more prevalent, with 4/14 (28.6%) cases involving both lungs and 2/14 (14.3%) cases involving multiple lobes in the right lung (Table 3 and E4). All infectious lesions were peripheral, whereas nine of the 45 NENs (20%) were central tumors (*p* = 0.02). In the infection group, 63% (17/27) of lesions had distal atelectasis or inflammatory exudation, whereas 66.7% (30/45) of NENs had no adjacent lung abnormalities (*p* = 0.001). Compared to NENs, infections were more likely to be accompanied by short-term dynamic changes. Among the 23 infectious lesions with follow-up, 12 (52.2%) first grew then diminished or resolved, five (21.7%) presented new lesions, four (17.4%) decreased, and two (8.7%) remained stable. However, LNETs were relatively stable or grew slowly during follow-up (Fig. 6).

### Functional imaging results

The positive detection rate of octreotide scintigraphy was 21.2% (7/33) in the NEN group and 8% (2/25) in the infection group (*p* = 0.28). The positive rates of the ^18^F-FDG PET/CT scans were 70.0% (28/40) and 76.9% (20/26) in NENs and infectious lesions, respectively (*p* = 0.59). The median SUV_max_ was 1.3 (0.9, 3.1) among 28 pulmonary NENs and 2.4 (1.2, 5.7) among 20 infectious lesions, showing a higher tendency of SUV_max_ in the infection group (*p* = 0.07). Regarding ^68^Ga-DOTATATE PET/CT, the NEN group was likely to show a higher positive rate than the infection group (6/13, 46.2% vs. 1/13, 7.7%, *p* = 0.07) (Table 2).

The positive rate of ^68^Ga-DOTATATE PET/CT was 46.2% (6/13) in the NENs group, which was lower than in the ^18^F-FDG PET/CT group (70.0%) (Table 2). Furthermore, we classified the NENs group into three subgroups for detailed analysis (Table E6). Patients with ^18^F-FDG PET/CT negative (FDG^−^, *n* = 12) NENs had a longer time from symptom onset to diagnosis (23.5 vs. 12.0 months, *p* = 0.01) and a higher delta CT value on the contrast CT (68.9 vs. 44.3 HU, *p* = 0.01) compared to their positive counterparts (FDG^+^, *n* = 28). NENs with negative ^68^Ga-DOTATATE PET/CT findings (Ga^−^, *n* = 7) presented with a lower serum potassium (2.7 vs. 3.3 mmol/L, *p* = 0.01) and pericardial effusion rate (14.3% vs. 83.3%, *p* = 0.03). In the subgroup that received both ^18^F-FDG and ^68^Ga-DOTATATE PET/CT scans (*n* = 12), four patients (33.3%) had double-PET/CT-negative (FDG^−^Ga^−^) results (Fig. 6 and E6), three patients (25%) had double-positive (FDG^+^Ga^+^) results, and five cases had either ^18^F-FDG positive or ^68^Ga-DOTATATE PET/CT positive results [three cases (25%) with FDG^+^Ga^−^ results and two cases (16.7%) with FDG^−^Ga^+^ results]. The FDG^−^Ga^−^ cases had a tendency for delayed diagnosis (25.5 vs 6.0 vs. 12.0 months, *p* = 0.05) and smaller tumor size (8.9 vs. 10.3 vs. 15 mm, *p* = 0.34, Fig. E6) compared to the FDG^+^Ga^−^/FDG^−^Ga^+^ and FDG^+^Ga^+^ cases. All FDG^−^Ga^−^ tumors were peripheral tumors located within 15 mm of the pleura and with a tendency for a higher delta CT value on the contrast CT (76.3 vs 62.7 vs. 24.1 HU, *p* = 0.09).

## Discussion

In this study, we summarized the clinical and radiological manifestations of ectopic adrenocorticotropic hormone syndrome in 45 patients with pulmonary neuroendocrine neoplasms and 14 patients with tumor-like pulmonary infections. To our knowledge, this is the first study to comprehensively analyze the CT imaging features of this rare disease in such a large sample size. The findings revealed that CT features can help effectively distinguish between neuroendocrine neoplasms and tumor-like infections in ectopic adrenocorticotropic hormone syndrome patients.

Most biochemical results, such as ACTH, cortisol, and other inflammatory indicators were not significantly different between the two groups. Infection in hypercortisolism may lack typical manifestations of infectious disease such as high temperature and white blood cell count [20]. Limited laboratory evidence of infection highlights the significance of radiological methods, particularly CT scan, in the differential diagnosis of EAS.

Chest CT scan is easily accessible and routinely used to diagnose EAS, due to the high prevalence of EAS originating in the lung. Our study showed that a chest CT scan was useful for identifying EAS lesions and distinguishing NENs from infections. First, multifocal peripheral lesions, cavity, spiculation, and pleural retraction on CT images indicate a pseudotumor-like infection in EAS cases. Opportunistic infections by *Nocardia* species and fungi tend to form cavities. Xu et al reported that cavity (6/12, 50%), consolidation/infiltration (5/12, 42%), and nodule/mass (4/12, 33%) were frequent imaging features in EAS with *Nocardia* infection [21]. Another study reported similar findings, stating that cavity (8/16) was the most common finding, followed by nodules (7/16), infiltration (3/16), and consolidation (2/16) [22]. Second, overlying vessel sign and airway involvement were more common in EAS NENs, which may be correlated with the abundant vessels and a common airway origin in well-differentiated NETs [23, 24]. Third, most NENs presented with moderate-to-intense enhancement, higher than the infection lesions, and had a moderate capacity to be distinguished (AUC = 0.774). This may be attributed to the large number of NETs (carcinoid) in this cohort (71.1% NET G1 and 20% NET G2) and the high vascularity of carcinoids leading to significant enhancement on CECT [25, 26]. High-grade NENs generally have a higher incidence compared to their low-grade counterparts [5]. However, in EAS cases, lower-grade NENs are more frequently observed [3, 10, 27]. Few SCLC cases with EAS were diagnosed, possibly due to the severity of the primary disease obscuring the features of CS. Although CT findings may differ, histopathological confirmation is still necessary in cases with EAS and pulmonary masses. Additionally, 80% of EAS NENs had a tumor size less than 2 cm in our study, which may be attributed to the application of thin-slice CT reconstruction [28] and early diagnosis due to severe hypercortisolism [29]. The tumor size of NENs leading to EAS (mean: 1.1 cm, range: 0.4–3.0 cm) [30] was reported to be smaller than non-functioning lung carcinoids (median: 2.1 cm, range: 0.7–9.0 cm) [31], which is consistent with our study (median [Q1, Q3], 1.1 [0.9, 1.7] cm). EAS NETs show distinct, more aggressive features compared with hormone-quiescent carcinoids, but the mechanism has not been clearly elucidated [30].

PET/CT is commonly used for localizing the source of EAS, however the sensitivity of ^68^Ga-DOTATATE PET/CT (46.2%) was lower than ^18^F-FDG PET/CT (70.0%) in our patients with NENs. Four EAS patients were false negative in both ^18^F-FDG and ^68^Ga-DOTATATE PET/CT, while CT successfully detected all small pulmonary NEN nodules. These were resected afterward with excellent postoperative results and complete resolution of symptoms. Thin-slice chest CT imaging offers better detection of small EAS lesions during breath-hold scans, whereas PET/CT performed during free breathing may cause respiratory motion artifacts and hamper the detectability of small lung lesions. The false negative rates in PET/CT may be attributed to well-differentiated EAS tumors with low uptake [32]. In EAS localization to the lungs, a previous study showed comparable sensitivity between ^68^Ga-SSTR PET/CT and CT [79.4% (7/9) vs 77.8% (77/97)] [32]. Another study reviewed ^68^Ga-PET/CT results in 69 EAS cases and revealed a slightly lower sensitivity compared to CT (64.0% vs 69.7%) [33]. False negative scans from ^68^Ga-SSTR PET/CT may be underreported. Ceccato et al also reported an EAS patient that was negative on ^68^Ga-SSTR-PET/CT but positive on ^18^F-FDG PET, which is likely attributed to tumor cell dedifferentiation and diminished expression of somatostatin receptors [34]. High cortisol level downregulates SSTRs expression, also influencing the accuracy of ^68^Ga-SSTR-PET/CT [35]. No significant elevation was observed in 24-h UFC in our double-negative PET/CT patients, but their tumor size tended to be smaller. A previous study consistently reported slightly smaller lesions (12 mm vs 16 mm, *p* = 0.26) in the ^68^Ga-SSTR PET/CT negative group [33].

Tumor-like infection in EAS can exhibit high uptake in PET/CT, leading to misdiagnosis as NENs. Hou et al reported a higher FDG uptake in pulmonary infectious lesions compared to EAS lung tumors (mean SUV_max_: 5.9 vs. 2.1, *p* = 0.01) [36]. In our study, the median SUV_max_ of ^18^F-FDG-PET/CT was also higher in pulmonary infections than in NENs, but with a borderline difference (2.4 vs. 1.3, *p* = 0.05). This discrepancy may be due to the different subtypes of infections and NENs. The majority of NENs in our study were NET G1 tumors, usually presenting with low FDG uptake [37]. *Cryptococcus* and *Candida* lesions had a high SUV_max_ (over 4.95), while lesions of *Nocardia*, *Aspergillus*, and other fungi presented with moderate FDG uptake. *Cryptococcus* nodules have been reported to exhibit increased ^18^F-FDG PET/CT uptake (SUV_max_= 13.6) [38, 39]. Therefore, functional imaging methods combined with CT scan are needed for precise diagnosis.

Our study had some limitations. First, the study population might be biased due to the rarity of EAS and the low availability of pulmonary infection cases occurring simultaneously. Second, this study was retrospective, and CECT scanning was conducted in only 78% of the patients. In addition, there is limited ^68^Ga-DOTATATE PET/CT data because this method has only been in use since 2012. Third, the patient numbers corresponding to each pathogen were relatively small; thus, larger sample sizes are required for further study.

In conclusion, this study comprehensively summarized the typical CT features of pulmonary neuroendocrine neoplasms and tumor-like infections in ectopic adrenocorticotropic hormone syndrome. Thin-slice CT images can provide a vivid evaluation of lesions and their relationship to adjacent lung tissues, such as small bronchi and vessels. Therefore, they can be valuable for localizing ACTH-secreting tumors, more prompt differential diagnosis, and effective treatment of EAS.

### Supplementary information

ELECTRONIC SUPPLEMENTARY MATERIAL

Video S1

## Data Availability

The data underlying this article are available in the article and the supplementary material.

## Abbreviations

ACTH: Adrenocorticotropic hormone
EAS: Ectopic adrenocorticotropic hormone syndrome
FDG: Fluorodeoxyglucose
LNETs: Lung NETs
NENs: Neuroendocrine neoplasms
NETs: Neuroendocrine tumors
