# Supplementary material for: Differentiating lung neuroendocrine neoplasms from tumor-like infection using CT in patients with ectopic ACTH syndrome
Source: Insights Imaging. 2024 Aug 1;15:187. doi: 10.1186/s13244-024-01775-9 (PMC11294316; doi:10.1186/s13244-024-01775-9)
Supplement: Supplementary file 1 — ELECTRONIC SUPPLEMENTARY MATERIAL [file 13244_2024_1775_MOESM1_ESM.pdf]

Differentiating Lung Neuroendocrine Neoplasms from Tumor-Like Infection Using CT in Patients with Ectopic ACTH Syndrome

ELECTRONIC SUPPLEMENTARY MATERIAL

Appendix E1

1.1 Study Inclusion and Exclusion Criteria

The inclusion criteria were as follows: (1) Confirmed diagnosis of ectopic adrenocorticotropic hormone (ACTH) syndrome (EAS); the EAS diagnosis flowchart is shown in **Figure E1**; (2) Having suspected pulmonary lesions on chest computed tomography (CT) images; (3) With detailed information of endocrinological, radiological, and/or pathological results; (4) In the case of EAS with pulmonary neuroendocrine neoplasms (NENs), the pathological results from surgery or biopsy were available and supported the diagnosis of EAS, and serum cortisol level decreased to < 5 μg/dL after surgical treatment; (5) in the case of EAS with pulmonary infection, the infection was verified by surgery or pathogenetic testing; (6) Availability of complete chest CT images; and (7) No radiotherapy or chemotherapy performed before nodule/mass resection. The exclusion criteria were as follows: (1) Incomplete medical information such as missing crucial biochemical data, hormone test results, radiological records, or insufficient follow-up data; (2) Motion or respiratory artifacts or diffuse pulmonary disease leading to poor CT image quality affecting nodule or mass observation on CT images.

1.2 1.2 Biological Evaluation for EAS

The cortisol and ACTH levels were measured by Siemens chemiluminescent immunoassays (Berlin, Germany). Endogenous hypercortisolism was first confirmed by overnight dexamethasone suppression test (ONDST) or 48-hour, 2 mg/day low-dose dexamethasone suppression test (LDDST). Normal responses consisted of a plasma cortisol level <1.8 μg/dl (50 nmol/L) on the ONDST, a 24-hour urinary free cortisol (UFC) level less than the lower limit of the normal range and/or a plasma cortisol level less than 1.8 μg/dl (50 nmol/L) on the LDDST[1]. The high-dose dexamethasone suppression test (HDDST) was conducted as 2 mg dexamethasone given orally every 6 hours for 48 hours or as a single 8 mg overnight dose of dexamethasone. The percentage of plasma cortisol or UFC suppression after dexamethasone administration was then measured. The failure of UFC and/or plasma cortisol suppression (less than 50%) supported a possible

diagnosis of EAS[1]. Bilateral inferior petrosal sinus sampling (BIPSS) was performed before and after the intravenous injection of desmopressin (DDAVP) 10 mg. This procedure is usually conducted in the differential diagnosis of ACTH-dependent Cushing's syndrome (CS) when the results of clinical, biochemical, and radiological tests are discordant[1]. The conditions included the following: (1) Negative findings on pituitary magnetic resonance imaging (MRI); (2) No suppression on the HDDST, but pituitary MRI indicated a pituitary lesion < 6 mm; and (3) Occasionally, lesions were present in both the pituitary and peripheral visceral organs such as the lungs, mediastinum and pancreas, which are common sources of EAS tumors. The ratio of the central to peripheral ACTH level of < 2 at baseline and < 3 after DDAVP administration indicated ectopic ACTH production.

1.3 Image Interpretation of Chest CT

The morphological features of each lesion and adjacent pulmonary parenchyma were assessed, including size (maximum long-axis diameter and perpendicular short-axis diameter), shape (round, oval, polygonal, or irregular), margins (smooth or non-smooth), density (pure ground-glass opacity, partial-solid or solid), mean CT attenuation, degree of CT enhancement, lesion location, cavity, calcification, adjacent pulmonary abnormality (vascular convergence sign, peripheral bronchiectasis, and pleural retraction sign), pleural effusion, pericardial effusion, and regional lymph node enlargement. The involvement of the lobar and airway (main airway, lobar, segmental or subsegmental bronchus) was also evaluated using coronal, sagittal and minimum intensity projection (MinIP) reconstruction images when necessary. Affected segmental or larger bronchi were defined as central lesions; peripheral lesions were defined when subsegmental and more distal bronchial involvement or tumors surrounded by lung parenchyma without direct airway involvement were found. Mean CT values were measured on a sufficiently large round or oval region of interest (ROI) within the lesion on the maximum axial section, avoiding the air-containing area, cystic space, or blood vessels as much as possible. The definitions of other CT features are described in **Table E1**. If previous or follow-up chest CT scans were available, the changes in lung lesions were also evaluated. If contrast-enhanced CT or CT pulmonary angiography were conducted, the conditions of pulmonary embolism were also evaluated. The quantitative measurements from the two radiologists were averaged, and the consensus on each qualitative evaluation was documented.

1.4 ^{18}F-FDG Positron Emission Tomography (PET)/CT protocol

PET/CT was performed on a Siemens Biograph 64 TruePoint scanner (Siemens Healthcare, Germany). The patients were asked to fast for eight hours so the blood glucose level was less than 120 mg/dL before the intravenous injection of 5.55 MBq/kg ^{18}F–FDG. Imaging acquisition was conducted 60 minutes after the ^{18}F–FDG administration. The attenuation correction CT (120 kV, 140 mA, and 4.25 mm collimation) scan was followed by a PET scan from the head to the mid-thigh in the three-dimensional mode at two minutes per bed position. The PET data were reconstructed using the ordered set expectation maximization algorithm method. CT images were used for attenuation correction of the PET data and anatomic localization. The co-registered images were then displayed on the workstation (Siemens Medical System) for evaluation. The images were reviewed by a senior nuclear medicine physician who was blinded to the pathological and pathogenetic testing results. A region of interest (ROI) was placed on the pulmonary lesion to measure the maximum standard uptake value (SUV_{max}) using the following formula: SUV_{max} = maximum pixel activity/(injected doses/body weight).

1.5 ^{68}Ga-DOTA-TATE PET/CT protocol

The ^{68}Ga-DOTATATE was conducted in accordance with previously published literature[2]. In brief, the research was conducted using a time-of-flight PET/CT scanner (Siemens Co.). The 111–148 MBq (3–4 mCi) ^{68}Ga-DOTATATE was administered intravenously to the patients. At 40–60 minutes after injection, a low-dose whole-body CT scan (120 keV; 100 mAs; 1.3 pitch; 2.5 mm slice thickness; 0.5 s rotation time; estimated radiation dose, 9.0 mGy) was obtained for anatomic localization and attenuation correction. PET scanning was performed at 1.5 minutes per bed position with a 23 slice overlap. Reconstructed images were corrected for CT-based attenuation, dead time, random events, and scatter using ordered-subset expectation maximization (2 iterations, 10 subsets, 192 × 192 matrix). The DOTATATE PET/CT scans were examined for regions of increased tracer uptake. The tracer uptake pattern and the SUV_{max} were recorded. PET positive was determined when the tracer uptake in the lesion was higher than the adjacent background.

1.6 Somatostatin receptor scintigraphy (SRS) protocol

^{99m}Tc-HYNIC-TOC was synthesized and labeled as previously described[3]. After intravenous administration of the tracer, whole-body images were acquired at 1 and 4 hours using a Hawkeye dual-head camera (GE Medical, Milwaukee, WI). When increased uptake in the chest was noted, patients underwent pulmonary SPECT/CT imaging. Two experienced nuclear medicine physicians who were not aware of the histopathologic diagnosis, or the previously reported interpretation of the scintigraphy findings interpreted the images jointly. Lesions with tracer uptake higher than the adjacent background were determined as positive.

Supplementary Tables

Table E1. The definitions and classifications for clinical characteristics, CT and functional imaging features

Feature	Definition	Unit or Classification
**Basic clinical features**		
Age	Age of patients	Unit, years old
Sex	Gender of patients	1, male; 2, female
Smoking history	The history of smoking cigarettes	0, non-smoker; 1-current smoker or former smoker
Course of disease	Time from symptoms onset to medical consultation	Unit, months
Time to diagnosis	Time from symptoms onset to confirmed diagnosis	Unit, months
Time of follow-up	Time from diagnosis onset to the latest follow-up	Unit, months
Clinical features of EAS	The clinical presentations including diabetes mellitus/ glucose intolerance, osteoporosis, hypertension, hypercortisolism, and hypopotassemia. Physical examination index including waistline and BMI. Biochemical analysis including serum potassium, preoperative ACTH, morning serum cortisol, 24-hour urinary free cortisol (UFC), total cholesterol, triglyceride, high-density lipoprotein and low-density lipoprotein.	
Localization tests of EAS	The high-dose dexamethasone suppression test (HDDST), bilateral inferior petrosal sinus sampling (BIPSS), pituitary MRI, somatostatin receptor scintigraphy (SRS), ^{18}F-fluorodeoxyglucose (FDG) or ^{68}Ga-DOTATATE-positron emission tomography (PET)/CT, adrenal gland CT were involved.	
Clinical features of infection	Clinical symptoms associated with infection including fever, cough, sputum. Laboratory tests including blood routine test, erythrocyte sedimentation rate (ESR), high-sensitivity C-reactive protein (hs-CRP), and etiological examination of sputum/blood/bronchoalveolar lavage fluid or biopsy specimen.	
**Histopathological features**		
Pathology (Histology subtype)	The type and grades of lung neuroendocrine neoplasms (NENs) were assessed according to the 2018 International Agency for Research on Cancer (IARC) and World Health Organization (WHO) classification of	1, Neuroendocrine tumor (NET) Grade 1 (G1); 2, NET G2; 3, Neuroendocrine carcinoma (NEC), Small cell

	NENs[4]. The diagnosis of EAS were verified by immunohistochemical staining for ACTH, Ki-67, CgA, Syn and occasionally CRH if the result for ACTH was negative. Pathogeny of lung infection were confirmed by surgery or etiological examination.	lung Carcinoma (SCLC); 4, aspergillus; 5, cryptococcus; 6, nocardiosis; 7, granulomatous inflammation or other fungi
	In the pulmonary NEN group, bronchus affected, pleural invasion, intravascular cancer thrombosis, or lymph node metastasis were also recorded.	0, no; 1, yes
**CT imaging features**		
Lesion type	Pulmonary nodule, small, rounded opacities within the lung with a diameter equal to or less than 30 mm; pulmonary mass, pulmonary opacification that measures more than 30 mm.	1, nodule; 2, mass
Maximal long-axis diameter (Max diameter)	Longest diameter of the maximum cross section with lung window. The average of measurements from two radiologists who independently evaluated the images was used as the final maximum diameter.	Unit, millimeter
Perpendicular short-axis diameter	The maximum short-axis diameter that measured perpendicular to the long-axis in the same plane. The average of measurements from two radiologists who independently evaluated the images was used as the final maximum short-axis diameter.	Unit, millimeter
Mean CT attenuation	Mean CT attenuation was measured by use of a region of interest (ROI) tool sized to encompass approximately two thirds of the long and short axes of the nodule/mass on the maximum axial section at mediastinal windows, avoiding the air-containing area, cystic space or blood vessels as far as possible. The average of measurements from two radiologists who independently evaluated the images was used as the final mean CT value.	Unit, Hounsfield unit (HU)
Degree of CT enhancement	Increased enhancement value means the CT attenuation values between non-contrast CT scan and contrast-enhanced CT. Degree of enhancement is divided as follows: slight, the increase is less than 30 HU; moderate, the increase is between 30 and 60 HU; obvious, the increase is more than 60 HU	0, no; 1, slight; 2, moderate; 3, obvious
Density	Pure ground-glass opacity, unmasking the bronchi and vascular branches below; partial solid, presence of ground-glass opacity and solid components; solid, absence of ground-glass opacity	1, pure ground-glass opacity (pGGO); 2, partial solid; 3, solid
Shape	Round or oval, no spiculation or lobulation or irregularity of lesion margin;	1, round or oval; 2, polygonal or irregular

	polygonal or irregular, appearance of polygons or irregularity of lesion margin	
Contour	Margin of the lesion	1, smooth; 2, non-smooth
Lobulation	Surface of the tumor showed a wavy or scalloped configuration	0, no; 1, shallow; 2, deep
Spiculation	Short lines radiating from tumor margin	0, no; 1, yes
Cavity	Thick-walled abnormal gas-filled spaces within the lung nodule/mass	0, no; 1, yes
Airway involvement	The relationship of lesion with adjacent airway were evaluated. Airway involvement was defined as present if a CT scan showed a component of the lesion either within the lumen of or obstructing the lumen of a visible airway.	1, main or lobar bronchi involved; 2, segmental or subsegmental involved; 3, no discernable airway involvement
Air bronchogram	Air bronchogram refers to the phenomenon of air-filled bronchi being made visible by the opacification of surrounding alveoli.	0, no; 1, yes
Vascular connections	Relationship of lesions and connected vascular.	1, no discernable vascular connection; 2, connected to pulmonary veins only; 3, connected to pulmonary arteries only; 4, connected to both pulmonary artery and pulmonary vein
Overlying vessel sign	The curved vascular shadow or sectional vascular shadow around the lesion can be seen on CT image especially on contrast-enhanced CT images.	0, no; 1, yes
Pleural retraction sign	Retraction of the pleura toward the nodule/mass	0, no; 1, yes
Lesion-pleura relationship	The distance and attachment relationship between lesion and adjacent pleura.	0, tightly attached; 1, ≤15mm; 2, >15mm
Distance from pleura	The distance from lesions to adjacent pleura.	Unit, millimeter
Lesion location	Central, tumor originated from the segmental or more proximal bronchi; peripheral, tumor originated from the subsegmental bronchi or more distal airway, or tumors surrounded by lung parenchyma without direct airway involvement	1, central; 2, peripheral
Lobe of lung involved	LLL, left lower lobe; LUL, left upper lobe; RLL, right lower lobe; RML, right middle lobe; RUL, right upper lobe; mixed, lesions across lobes; BM, bilateral multiple lesions	1, LLL; 2, LUL; 3, RLL; 4, RML; 5, RUL; 6, mixed; 7. BM

Pulmonary embolism	Pulmonary embolism was identified.	0, no; 1, yes; 2, evaluation not available with non-contrast enhanced CT scan
Lymph node enlargement	Enlarged local lymph nodes (hilar or mediastinal) with short-axis diameter greater than 1 cm	0, no; 1, yes
Pleural effusion	Effusion seen in the thorax.	0, no; 1, yes
Pericardial effusion	Effusion seen in the pericardial cavity.	0, no; 1, yes
Adjacent pulmonary abnormalities	Pulmonary abnormalities adjacent or distal to the lesion	0, no discernable abnormality; 1, accompanied by other nodules; 2, hyperlucency with or without bronchiectasis; 3, distal atelectasis or inflammatory exudation
PET-CT and SRS characteristics		
SUV_{max} of ^{18}F-FDG PET/CT	Maximum ^{18}F-FDG standard uptake value in the region of interest placed on the primary tumor. It is calculated as: SUV_{max} = maximum pixel activity/(injected doses/body weight)	Unit, none
SUV_{max} of ^{68}Ga-DOTATATE PET/CT	Maximum ^{68}Ga-DOTATATE standard uptake value in the region of interest placed on the primary tumor. SUV_{max} was calculated by measuring the maximum concentration of the labeled tracer in the lesion divided by the decay-corrected injected activity and normalized for body weight.	Unit, none
Results of SRS	The results of somatostatin receptor scintigraphy (SRS) were noted as either positive or negative within the primary lesion.	0, negative; 1, positive

Table E2. Parameters of CT scanners and reconstruction methods

Parameters		**** Hospital				
**CT system information**	CT scanner system	Dual Source CT (Siemens Healthcare, Germany)		Spectral CT (Discovery CT750 HD scanner, GE Medical Systems, USA)	Spectral CT (IQon CT, PHILIPS, Netherlands)	64-channel CT (Aquilion 64 CT, Toshiba, Japan)
		Somatom Definition Flash	Somatom Force			
**Number of patients**	Number of patients	21	18	11	5	4
**CT scan parameters**	Tube voltage	120 kVp		120kVp	120kVp	120kVp
	Tube current	Variable tube current with automatic tube-current modulation activated		Variable tube current with automatic tube-current modulation activated	Variable tube current with automatic tube-current modulation activated	Variable tube current with automatic tube-current modulation activated
	Rotation time	0.5s		0.6 s	0.5s	0.5s
	Detector collimation	64×0.6 mm		64×0.625 mm	64×0.625 mm	64×0.625 mm
	Pitch	1.2		0.984	1.2	0.984
	Arterial phase	35s after injection		35s after injection	35s after injection	35s after injection
	Image matrix	512×512		512×512	512×512	512×512
	Field of view	350×350 mm		350×350 mm	350×350 mm	350×350 mm
	Reconstruction slice thickness/slice increment	1mm/1mm		0.625mm/0.625mm	1mm/1mm	1mm/1mm

Reconstruction algorithm	standard resolution/lung	standard resolution/lung	standard resolution/lung	standard resolution/lung
Three-dimensional post-processing method	Coronal/Sagittal/Minimum density reconstruction if necessary	Coronal/Sagittal/Minimum density reconstruction if necessary	Coronal/Sagittal/Minimum density reconstruction if necessary	Coronal/Sagittal/Minimum density reconstruction if necessary

Table E3. Laboratory indicators of inflammation, tumor, serum lipid, blood routine examination, and myocardial enzymes between EAS NEN and pulmonary infections

	**EAS with pulmonary NENs**	**EAS with pulmonary infection**	***P***
**Number of patients**	45	14	
ESR (mm/h)	7.0 (4.0, 13.0)	18.0 (6.5, 67.8)	0.059
hs-CRP (mg/L)	0.8 (0.5, 1.7)	14.2 (5.8, 25.6)	< 0.001
Cyfra211 (ng/ml，≤3.5)	4.0 (2.9, 6.8)	5.3 (4.3, 5.6)	>0.05
SCCAg (ng/ml，≤2.7)	0.8 (0.6, 1.5)	0.7 (0.6, 1.0)	>0.05
AFP (ng/ml，≤20)	3.9 (2.5, 4.7)	3.5 (2.6, 4.9)	>0.05
ProGRP (pg/ml，≤69.2)	77.5 (37.8, 129.8)	49.0 (29.2, 88.7)	>0.05
NSE (ng/ml，≤16.3)	13.0 (11.1, 16.0)	12.6 (11.5, 13.8)	>0.05
CEA (ng/ml，≤5)	4.4 (3.3, 6.3)	3.6 (2.6, 5.9)	>0.05
CA72-4 (U/ml，≤9.8)	2.4 (1.5, 9.7)	2.0 (1.9, 8.2)	>0.05
CA242 (U/ml，≤20)	6.6 (5.4, 12.1)	8.3 (4.8, 9.6)	>0.05
CA19-9 (U/ml，≤34)	22.6 (13.9, 48.6)	24.8 (15.9, 64.1)	>0.05
CA15-3 (U/ml，≤25)	11.7 (8.7, 16.5)	13.4 (13.0, 20.7)	>0.05
CA125 (U/ml，≤35)	25.2 (15.5, 38.8)	40.6 (12.3, 48.9)	>0.05
Gastrin (pg/ml)	34.7 (24.0, 59.4)	39.7 (20.0, 64.8)	>0.05
Fungal D-glucan G test (pg/ml, <100.5)	50.0 (15.3, 79.5)	30.9 (21.0, 237.3)	>0.05
TC (mmol/L)	5.1 (4.0, 6.2)	4.9 (3.7, 5.2)	>0.05
TG (mmol/L)	1.6 (1.1, 2.1)	1.7 (1.2, 2.2)	>0.05
HDL-C(mmol/L)	1.3 ± 0.4	1.1 ± 0.3	>0.05
LDL-C (mmol/L)	3.0 ± 1.2	2.9 ± 1.4	>0.05

WBC(*10^9/L)	8.4 ± 2.7	9.4 ± 2.7	>0.05
Lymphocyte (%)	12.6 (9.6, 17.6)	10.7 (5.9, 27.2)	>0.05
Monocyte (%)	5.5 ± 1.8	4.4 ± 1.6	>0.05
Neutrophil (%)	80.4 (73.2, 84.9)	84.3 (64.3, 90.2)	>0.05
Platelet (*10^9/L)	200.5 (160.0, 257.5)	176.5 (150.8, 248.3)	>0.05
Creatine kinase (CK, U/L)	67.0 (36.0, 123.5)	41.5 (32.8, 72.3)	>0.05
Creatine kinase MB mass (CK-MB mass, *μ*g/L)	1.6 (0.8, 2.3)	1.3 (0.8, 1.8)	>0.05
Cardiac troponin (cTnI, *μ*g/L)	0.02 (0, 0.05)	0.02 (0.01, 0.02)	>0.05
N-terminal B-type natriuretic peptide (NT-proBNP,pg/mL)	346.0 (75.0, 662.8)	285.5 (142.3, 602.0)	>0.05

Abbreviations: EAS, Ectopic adrenocorticotropin syndrome; ESR, erythrocyte Sedimentation Rate; hs-CRP, high-sensitivity C-reactive protein; SCCAg, squmaous cell carcinoma antigen; AFP, alpha fetoprotein; ProGRP, gastrin releasing peptide precursor; NSE, neuron-specific enolase; CEA, carcinoma embryonic antigen; TC, total cholesterol; TG, triglyceride; HDL-C, high-density lipoprotein cholesterol; LDL-C, low-density lipoprotein cholesterol; WBC, white blood cell.

Table E4. Radiological characteristics of 27 tumor-like pulmonary infection in 14 EAS cases

Patient	Size（mm）	Lesion location	Number of nodules	Distance from pleura (mm)	Shape	Spiculation	Pleural retraction sign	Overlying vessel sign	Cavity	Enhancement (HU)
1	13	LLL	single	12	Polygonal or irregular	-	-	-	+	NA
2	25	RLL	single	0	Polygonal or irregular	-	-	-	+	NA
3	11	RLL	multiple	1	Round or oval	-	-	-	-	48
	5	LUL		19	Round or oval	-	-	-	-	48
	5	LUL		21	Polygonal or irregular	-	-	-	-	34
4	30	RLL	multiple	4	Polygonal or irregular	+	+	+	+	10
	11	RML		0	Round or oval	-	+	+	-	40
5	80	RUL	single	0	Polygonal or irregular	-	+	-	+	NA
6	5	RML	single	11	Round or oval	-	-	-	-	27
7	16	LUL	multiple	13	Polygonal or irregular	+	-	+	+	10
	45	RUL		0	Polygonal or irregular	+	+	+	+	20
	40	RUL		0	Round or oval	+	+	-	+	21

8	16	RML	single	0	Round or oval	-	-	-	+	53
9	5	RUL	multiple	9	Round or oval	-	-	-	-	18
	6	RUL		5	Round or oval	-	-	-	-	8
	10	RLL		5	Round or oval	-	+	-	-	6
10	9	RML	single	0	Polygonal or irregular	-	+	-	+	41
11	39	RUL	multiple	7	Polygonal or irregular	-	-	-	+	14
	15	LUL		3	Round or oval	-	+	-	-	11
	14	RUL		3	Round or oval	+	+	-	+	16
12	9	LUL	multiple	15	Polygonal or irregular	-	+	-	+	NA
	39	RUL		7	Polygonal or irregular	+	+	-	+	NA
	16	RUL		8	Polygonal or irregular	-	+	-	+	NA
13	15	RUL	single	4	Polygonal or irregular	-	+	-	+	NA
14	8	LLL	multiple	6	Round or oval	-	-	+	-	4
	12	LLL		7	Round or oval	-	-	-	-	3
	8	LLL		17	Round or oval	-	-	-	-	37

Insights Imaging (2024) Song L, Miao H, Zhu Zc, et al.

Abbreviations: EAS, Ectopic adrenocorticotropin syndrome; LLL, left lower lobe; LUL, left upper lobe; RLL, right lower lobe; RML, right middle lobe; RUL, right upper lobe; NA, not available.

Table E5. Radiological characteristics of 45 pulmonary NENs in 45 EAS cases

Patient	Pathology	Size（mm）	Lesion location	Number of nodules	Distance from pleura (mm)	Shape	Spiculation	Pleural retraction sign	Overlying vessel sign	Cavity	Enhancement (HU)
1	NET G1	36	LUL	single	8	Round or oval	-	-	+	-	NA
2	NET G1	15	LLL	single	18	Round or oval	-	-	+	-	4
3	NET G1	10	LUL	single	18	Round or oval	-	-	+	-	61
4	NET G2	16	RML	single	2	Polygonal or irregular	+	+	+	-	37
5	NET G1	5	RML	single	6	Round or oval	-	-	+	-	28
6	NET G1	9	LUL	single	14	Round or oval	-	-	+	-	18
7	NET G2	19	RLL	single	0	Round or oval	-	-	+	-	NA
8	SCLC	39	RLL	single	0	Polygonal or irregular	-	-	-	-	NA
9	NET G2	15	RML	single	15	Round or oval	-	-	+	-	57
10	NET G1	6	LLL	single	17	Round or oval	-	-	-	-	68
11	NET G1	10	RLL	single	12	Round or oval	-	-	+	-	41
12	SCLC	32	RUL	multiple	0	Round or oval	-	+	+	-	37
13	NET G1	10	LLL	single	13	Round or oval	-	-	+	-	17
14	NET G1	10	RML	single	11	Round or oval	-	-	+	-	NA
15	NET G1	15	RUL	single	0	Polygonal or irregular	-	-	+	-	16
16	NET G1	8	RML	single	5	Round or oval	-	-	+	-	NA
17	NET G1	13	RLL	single	0	Round or oval	-	-	+	-	NA
18	NET G1	5	LUL	single	12	Round or oval	-	-	+	-	NA
19	NET G2	13	RML	single	0	Round or oval	-	-	-	-	72
20	NET G1	3	RUL	single	12	Round or oval	-	-	-	-	6
21	NET G1	9	RML	single	0	Round or oval	-	-	+	-	93
22	NET G1	9	RUL	single	17	Polygonal or irregular	-	-	+	-	48
23	NET G2	11	RLL	single	11	Round or oval	-	-	+	-	65
24	NET G1	11	RML	single	14	Round or oval	-	-	+	-	65
25	NET G1	23	RLL	single	0	Polygonal or irregular	-	-	+	-	14
26	NET G1	9	LLL	single	0	Round or oval	-	-	-	-	32
27	NET G1	10	LLL	single	0	Round or oval	-	-	+	-	2

28	SCLC	32	LUL	multiple	0	Polygonal or irregular	-	+	-	-	NA
29	NET G1	8	LLL	single	2	Round or oval	-	-	-	-	119
30	NET G1	8	LUL	single	4	Round or oval	-	-	-	-	76
31	SCLC	25	Parahilar (mixed)	single	0	Polygonal or irregular	-	-	+	-	15
32	NET G1	10	LLL	multiple	3	Round or oval	-	-	-	-	85
33	NET G1	21	LUL	single	0	Polygonal or irregular	-	-	+	-	51
34	NET G2	23	LUL	single	0	Polygonal or irregular	-	-	+	-	53
35	NET G1	10	LLL	single	3	Polygonal or irregular	-	-	-	-	51
36	NET G2	15	RML	single	0	Polygonal or irregular	-	-	+	-	38
37	NET G2	7	LUL	single	13	Round or oval	-	-	+	-	37
38	NET G1	10	RML	single	10	Round or oval	-	-	-	-	165
39	NET G2	6	RLL	multiple	1	Round or oval	-	-	-	-	82
40	NET G1	13	LUL	single	0	Round or oval	-	-	-	-	25
41	NET G1	10	LUL	single	0	Round or oval	-	-	-	-	53
42	NET G1	11	RML	single	0	Round or oval	-	-	+	-	53
43	NET G1	12	RML	single	0	Round or oval	-	-	+	-	49
44	NET G1	46	RLL	single	10	Polygonal or irregular	-	-	+	-	87
45	NET G1	39	RUL	multiple	7	Round or oval	-	-	-	-	75

Abbreviations: NETs: neuroendocrine tumors; SCLC, small cell lung cancer.

Table E6. Comparison of clinical and CT features in NENs with different PET/CT results

Items	**^{18}F-FDG PET/CT** (n=40)			**^{68}Ga-DOTATATE PET/CT** (n=13)			**^{18}F-FDG & ^{68}Ga-DOTATATE PET/CT** (n=12)			
	FDG$^+$	FDG$^-$	*P*1 value	Ga$^+$	Ga$^-$	*P*2 value	FDG$^-$Ga$^-$	FDG$^+$Ga$^-$ / FDG$^-$ Ga$^+$	FDG$^+$Ga $^+$	*P*3 value
**Number of patients**	28	12		6	7		4	5	3	
**Gender**			1.000			1.000				1.000
male	14 (50%)	6 (50%)		3 (50%)	3 (42.9%)		2 (50%)	2 (40%)	2 (67%)	
female	14 (50%)	6 (50%)		3 (50%)	4 (57.1%)		2 (50%)	3 (60%)	1 (33%)	
**Age (years)**	39.9 ± 20.0	42.8 ± 11.3	0.560	34.3 ± 17.7	43.4 ± 19.8	0.405	44.5 ± 13.2	39.8 ± 21.6	22.0 ± 3.6	0.228
**Duration of symptoms (months)**	17.5 (5.5, 27)	27.0 (12.8, 108.0)	0.055	12.0 (12.0, 14.0)	15.0 (8.5, 25.5)	0.489	25.5 (17.5, 44.5)	6.0 (4.0, 12.0)	14.0 (13.0, 20.0)	0.070
**Time from symptoms onset to diagnosis (months)**	12.0 (3.5, 20.0)	23.5 (12.8, 42.0)	**0.014**	12.0 (6.0, 12.0)	15.0 (9.0, 25.5)	0.255	25.5 (17.5, 44.5)	6.0 (5.0, 12.0)	12.0 (9.0, 13.0)	0.052
**Clinical features**										
Hypertension	25 (92.6%)	9 (75%)	0.159	5/5 (100.0%)	5/6 (83.3%)	1.000	3/4 (75.0%)	4/4 (100%)	3/3 (100%)	1.000
Diabetes mellitus/ glucose intolerance	19 (76.0%)	9 (90.0%)	0.644	3/4 (83.3%)	5/6 (83.3%)	1.000	4/4 (100.0%)	2/3 (66.7%)	2/3 (66.7%)	0.467
Osteoporosis	13 (92.9%)	8 (100.0%)	1.000	4/4 (100.0%)	4/4 (100.0%)	1.000	3/3 (100.0%)	2/2 (100.0%)	3/3 (100%)	1.000
Hypercortisolism	22 (78.6%)	10 (83.3%)	1.000	6/6 (100.0%)	6/7 (85.7%)	1.000	4/4 (100.0%)	4/5 (80.0%)	3/3 (100%)	1.000
Hypopotassemia	24 (85.7%)	11 (91.7%)	1.000	4/6 (66.7%)	7/7 (100%)	0.192	4/4 (100.0%)	4/5 (80.0%)	2/3 (66.7%)	0.697
**BMI (kg/m2)**	25.8 ± 3.6	25.0 ± 2.4	0.149	26.3±3.0	25.2±1.6	0.424	24.3 ± 2.0	26.5 ± 3.0	25.1 ± 0.1	0.458
**Laboratory tests of EAS**										
Preoperative ACTH (pg/mL)	152.5 (99.1, 254.8)	163.5 (99.0, 220.3)	0.988	140.75 (103.57, 170.88)	162.4 (141.85, 227.5)	0.445	187.7 (146.6, 227.5)	153.0 (87.8, 164.5)	117.0 (108.1, 149.5)	0.699

Morning serum cortisol (μg/dl)	48.0 (31.5, 75.0)	58.8 (36.7, 62.4)	0.988	56.64 (36.44, 62.755)	58.77 (36.585, 64.25)	0.945	60.0 (50.2, 139.0)	60.8 (31.5, 63.4)	31.1 (30.5, 41.8)	0.361
24-h UFC (μg/24h)	1513.2 (981.3, 2427.6)	1699.7 (1120.2, 2169.6)	0.959	1490.4 (1213.2, 1709.2)	1025.2 (706.85, 1481.9)	0.530	1367.0 (529.0, 3400.6)	1071.0 (1025.2, 1213.2)	2521.7 (543.4, 4500.0)	0.980
Serum potassium (mmol/L)	2.8 ± 0.8	2.5 ± 0.8	0.250	3.3 (3.3, 3.4)	2.7 (2.1, 2.8)	**0.007**	2.4 ± 0.5	2.9 ± 0.6	3.3 ± 0.1	0.190
**HDDST unsuppressed patients (%)**	15/27 (55.6%)	6/10(60.0%)	1.000	3/6(50%)	4/5(80%)	0.545	1/2(50.0%)	4/5 (80%)	1/3(33.3%)	0.714
**SRS (Octreotide scan) positive lesions**	5/20 (25%)	1/11 (9.1%)	0.383	1/5(20%)	1/6(16.7%)	1.000	0	1/5 (20%)	1/3 (33.3%)	1.000
**Lobar location of pulmonary lesions**			1.000			1.000				0.887
Left upper lobe	6 (22.2%)	3 (25%)		1(17%)	1(14%)		1 (25%)	1 (20%)	0	
Left lower lobe	6 (22.2%)	2 (16.7%)		2 (33%)	3(43%)		2 (50%)	1 (20%)	2 (66.7%)	
Right upper lobe*	4 (14.8%)	1 (8.3%)		0	0		0	0	0	
Right middle lobe	7 (25.9%)	4 (33.3%)		2 (33%)	2 (29%)		0	2 (40%)	1 (33.3%)	
Right lower lobe	4 (14.8%)	2 (16.7%)		1(17%)	1(14%)		1 (25.0%)	1 (20%)	0	
**Pericardial effusion**	14 (50%)	4 (33.3%)	0.332	5 (83.3%)	1 (14.3%)	**0.029**	1 (25.0%)	1 (20%)	3 (100%)	0.116
**Pleural effusion**	7 (25%)	1 (8.3%)	0.396	2 (33.3%)	1 (14.3%)	0.559	0	1 (20%)	1 (33.3%)	0.697
**Location of pulmonary lesions**			0.396			1.000				
Peripheral	21 (75%)	11 (91.7%)		6 (100%)	6 (86%)		4 (100%)	4 (80%)	3 (100%)	1.000
Central	7 (25%)	1 (8.3%)		0	1 (14%)		0	1 (20%)	0	
**Lesion-pleura relationship**			0.269			0.339				0.309
Tightly attached	11 (39.3%)	3 (25%)		2 (33.3%)	1 (14.3%)		0	1 (20%)	1 (33.3%)	
≤15mm from adjacent pleura	13 (46.4%)	9 (75%)		3 (50%)	6 (85.7%)		4 (100%)	4 (80%)	1 (33.3%)	
>15mm from adjacent pleura	4 (14.3%)	0 (0%)		1 (16.7%)	0		0	0	1 (33.3%)	

Insights Imaging (2024) Song L, Miao H, Zhu Zc, et al.

**Distance from pleura (mm)**	6.5 (0, 12.8)	2.6 (0, 6.3)	0.413	5.6 ± 7.1	2.2 ± 2.1	0.306	1.5 (0.5, 2.6)	4.0 (3.2, 6.0)	1.5 (0.8, 9.8)	0.316
**Maximum long-axis diameter (mm)**	10.6 (10.0, 15.1)	9.2 (8.0, 11.1)	0.112	13.2 (9.2, 15.8)	9.8 (7.2, 10.2)	0.116	8.9 (7.2, 9.9)	10.3 (8.0, 14.8)	15.0 (11.8, 15.5)	0.335
**Mean CT attenuation on non-contrast CT (HU)**	37.4 ± 17.3	27.9 ± 13.9	0.096	46.9 ± 21.8	27.3 ± 17.8	0.102	24.9 ± 18.8	31.5 ± 16.7	49.1 ± 24.3	0.295
**Delta CT value (HU)**	44.3 ± 34.4	68.9 ± 29.0	**0.010**	48.0 ± 30.6	65.1 ± 31.7	0.346	76.3 ± 31.7	62.7 ± 27.8	24.1 ± 18.0	0.086
**Lobulation**			0.287			1.000				0.879
None	15 (53.6%)	10 (83.3%)		5 (83.3%)	5 (71.4%)		3 (75%)	4 (80%)	2 (66.7%)	
Shallow	6 (21.4%)	1 (8.3%)		0 (0%)	1 (14.3%)		1 (25%)	0	0	
Deep	7 (25%)	1 (8.3%)		1 (16.7%)	1 (14.3%)		0	1 (20%)	1 (33.3%)	
**Pleural retraction sign**	3 (10.7%)	0	0.541	1 (16.7%)	0	0.462	0	0	1 (33.3%)	0.250
**Overlying vessel sign**	20 (71.4%)	5 (41.7%)	0.091	4 (66.7%)	2 (28.6%)	0.286	0	3 (60%)	2 (66.7%)	0.154
**Airway involvement**			0.603			1.000				0.758
Main or lobar bronchi involved	8 (28.6%)	2 (16.7%)		1 (16.7%)	2 (28.6%)		1 (25%)	1 (20%)	0	
Segmental or subsegmental bronchi involved	16 (57.1%)	9 (75%)		4 (66.7%)	4 (57.1%)		2 (50%)	4 (80%)	2 (66.7%)	
No discernable airway involvement	4 (14.3%)	1 (8.3%)		1 (16.7%)	1 (14.3%)		1 (25%)	0	1 (33.3%)	
**Adjacent pulmonary abnormality**			1.000			1.000				1.000
No discernable abnormality	19 (67.9%)	9 (75%)		5 (83.3%)	4 (57.1%)		3 (75%)		3 (100%)	
Accompanied by other nodules	1 (3.6%)	0		0	1 (14.3%)		0		0	
Hyperlucency/emphysema with or	2 (7.1%)	0		0	1 (14.3%)		0		0	

without bronchiectasis						
Distal atelectasis or inflammatory exudation	6 (21.4%)	3 (25%)	1 (16.7%)	1 (14.3%)	1 (25%)	0

Note: * One patient with the FDG-positive NEN had multiple lobes involvement of right lung, thus not included in the analysis.

Delta CT value: CT value increment after contrast-enhancement.

Abbreviations: BMI, body mass index; EAS, Ectopic adrenocorticotropin syndrome; ACTH, adrenocorticotropic hormone; UFC, urinary free cortisol; HDDST, high-dose dexamethasone suppression test; SRS, somatostatin receptor scintigraphy; FDG, fluorodeoxyglucose; PET-CT, - positron emission tomography-computed tomography.

Supplementary Figures

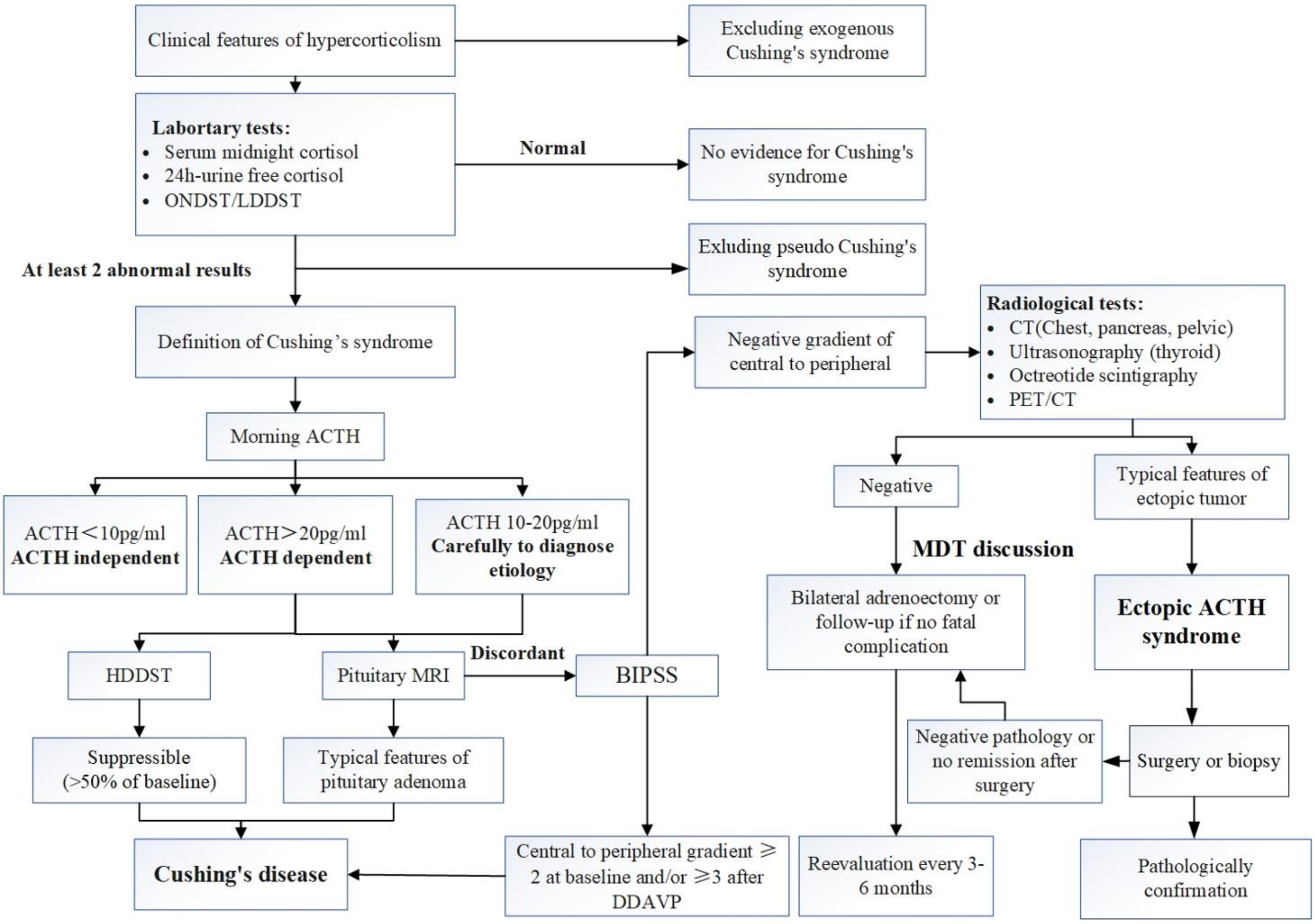

Figure E1. Diagnostic Flowchart of Ectopic Adrenocorticotropic Syndrome.

Abbreviations: ONDST, overnight dexamethasone suppression test; LDDST, low-dose dexamethasone suppression test; HDDST, high-dose dexamethasone suppression test; MRI, magnetic resonance imaging; BIPSS, bilateral inferior petrosal sinus sampling; DDAVP, desmopressin; CT, computed tomography; PET/CT, positron emission tomography/CT; MDT, multidisciplinary discussion.

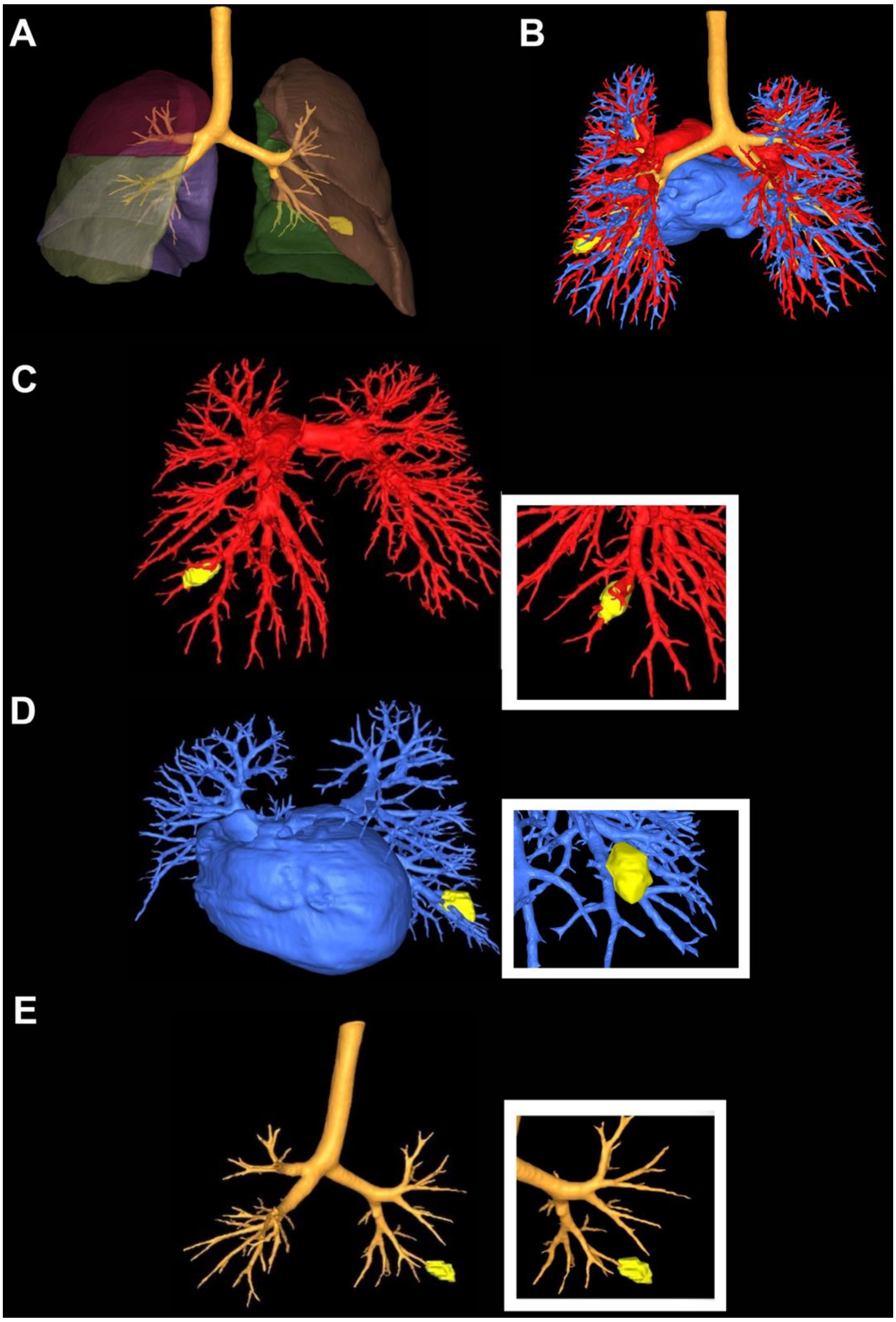

Figure E2. Coronal 3D Reconstruction/Surface Views of the Lung, Nodule and Bronchovascular Tree of a 21-year-old Man with Typical Carcinoid Tumor and Associated Ectopic Cushing's Syndrome (A-E). Lung lobes are depicted semitransparent (A), the nodule in left lower lobe is depicted in yellow (A-E), the bronchial tree is depicted in earth-yellow (A, B, E), the tree of pulmonary artery is depicted in red (B, C), and the tree of pulmonary vein is depicted in blue

(B, D). 3D reconstruction images of chest CT showed a peripheral irregular solid nodule in left lower lobe (A-E) with close relation to adjacent blood vessels (especially pulmonary artery, C) and bronchi (airway involvement, E).

Video S1 of the same patient was provided as Supplemental Material.

Abbreviations: 3D, Three-dimensional.

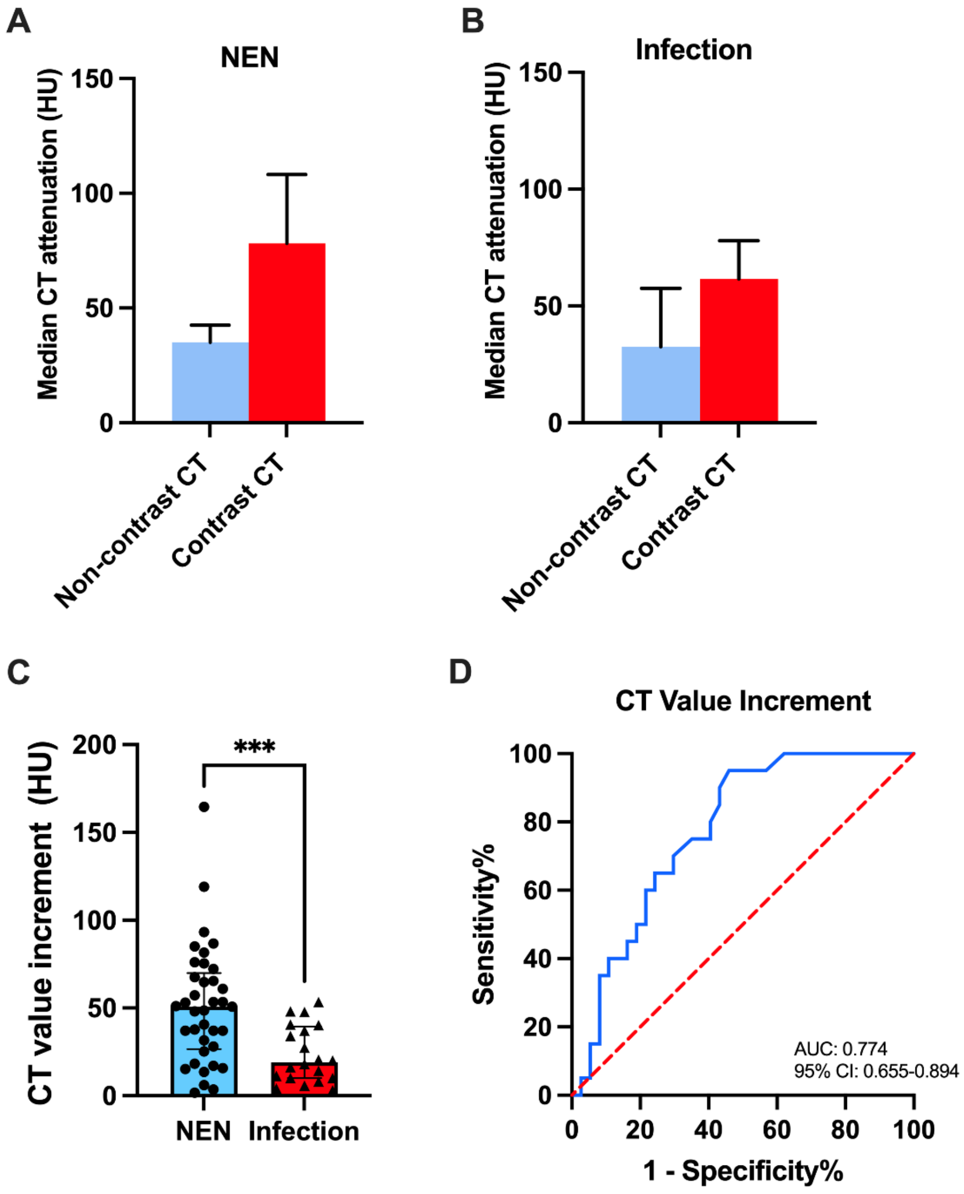

Figure E3. CT values of pulmonary neuroendocrine neoplasms and pulmonary infection in patients with ectopic ACTH syndrome. Fifty-seven lesions (37 NENs and 20 tumor-like infections) received both non-contrast CT and contrast-enhanced CT scans **(A, B)**. The median CT value increment between non-contrast and arterial phase (delta CT value) was significantly higher in NENs group than that in the infection group **(C)**. A receiver operating characteristic curve of CT value increment for differentiating pulmonary neuroendocrine neoplasms from tumor-like infections **(D)**. The area under the curve (AUC) is 0.774 (95% confidence interval [CI] 0.655-0.894; *P*<0.001). ***

P<0.001

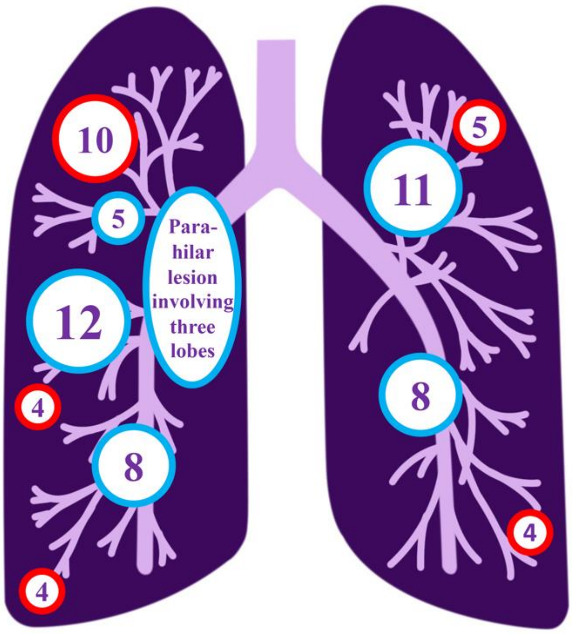

Figure E4. Schematic Diagram Showing Distribution tendency of NEN Group and Infection Group with EAS. The numbers in the red circle represent the number of lesions in each lung lobe of the infection group, and the numbers in the blue circle represent the number of lesions in each lung lobe of the NEN group. There is a para-hilar central lesion involving three lobes in NEN group, which is small cell lung cancer pathologic. NEN is more prevalent in right middle lobe and left upper lobe, while infection is more common in right upper lobe.

Abbreviations: EAS, Ectopic Adrenocorticotropic Syndrome; NEN, neuroendocrine neoplasm.

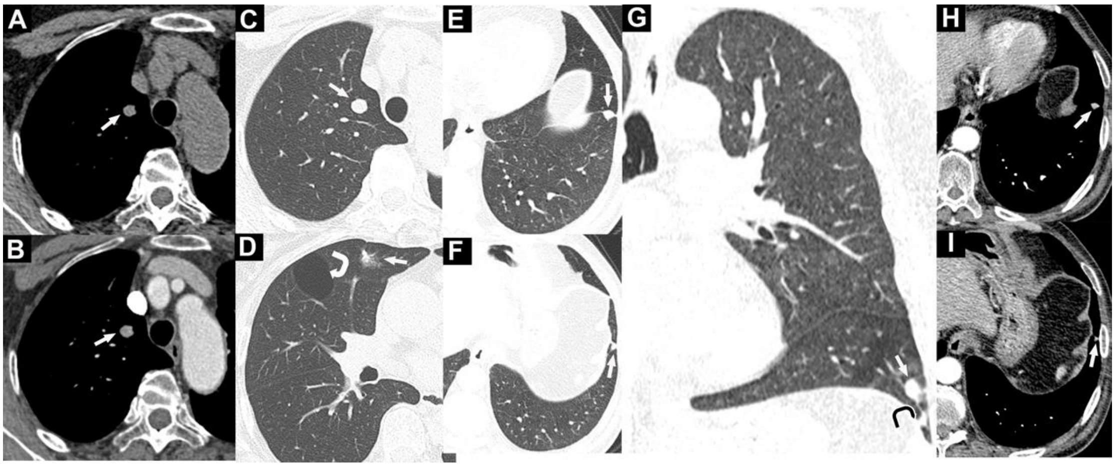

Figure E5. Two representative cases of lung neuroendocrine tumors accompanied by pulmonary nodules due to other causes. A-D) A 72-year-old woman with typical carcinoid tumor and associated ectopic Cushing's syndrome. Non-contrast (A) and contrast-enhanced (B) at mediastinal window, and non-contrast 1mm-thin-slice CT at lung window (C) showed a peripheral lobulated solid nodule (diameter 1.0cm) in the right upper lobe (RUL) (arrow), with moderate homogeneous enhancement (55HU increments). Non-contrast axial CT at lung window (D) showed another subpleural lobulated sub-solid nodule (diameter 1.4cm) in the RUL (arrow), which was invasive adenocarcinoma confirmed pathologically, accompanied by an adjacent pulmonary bulla (curved arrow). (E-I) A 68-year-old man with typical carcinoid tumor and associated ectopic Cushing's syndrome. 18F-FDG PET/CT showed a small solid nodule in the left lower lobe (LLL) with mild FDG activity (SUVmax=1.7), while 68Ga-DOTATATE PET/CT showed no increased uptake in the LLL nodule. And no obvious abnormality was found in somatostatin receptor scintigraphy. E, G, H) Chest CT showed a peripheral solid nodule (diameter 0.8cm) in the LLL (arrows), with significant homogeneous enhancement of 85HU increments (H), and pathological result was typical carcinoid with ACTH positivity in the immunohistochemistry test. Another micro solid nodule (arrow in F, curved arrow in G) next to the carcinoid nodule was also found on CT image with slight enhancement (arrow

in I), which was an intrapulmonary lymph node pathologically.

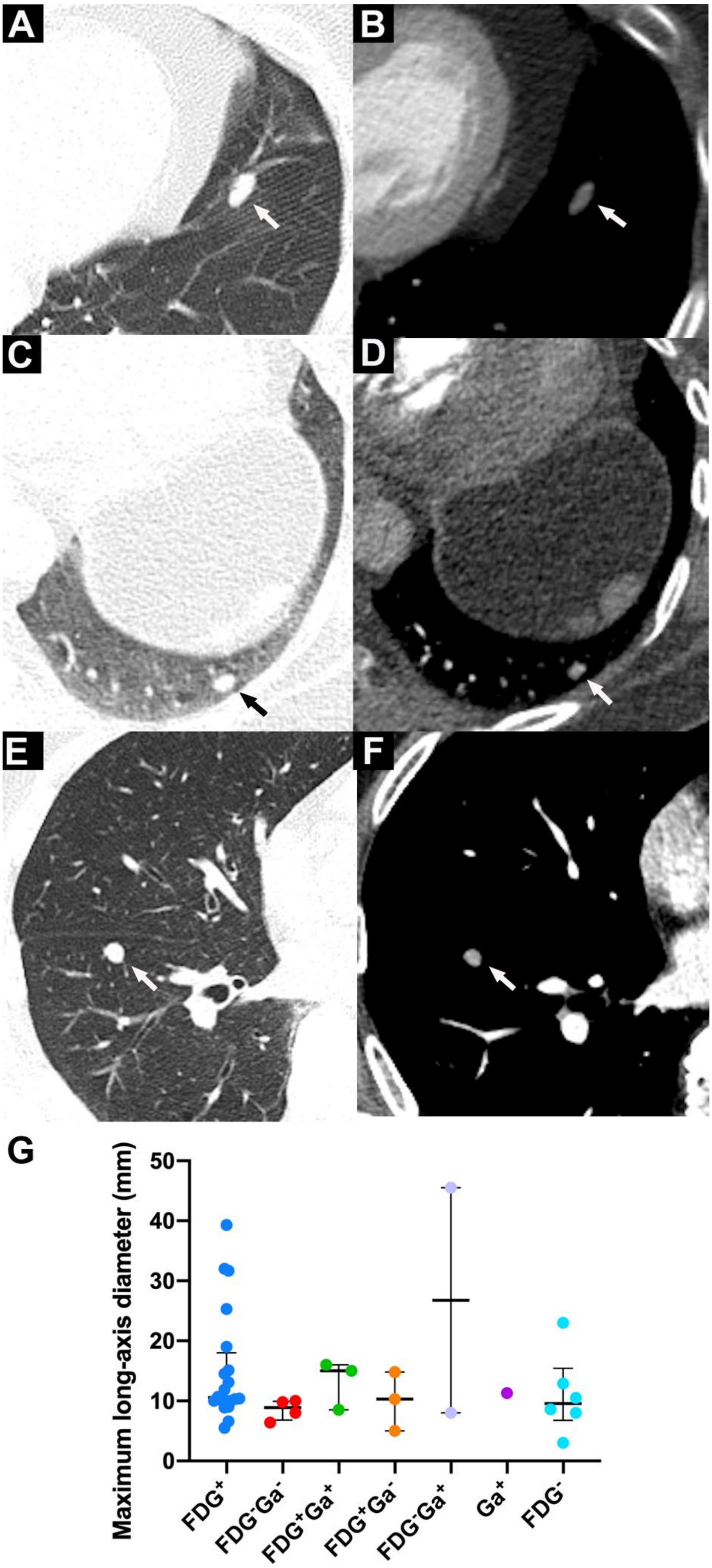

Figure E6. Three cases of lung neuroendocrine tumors that caused ectopic Cushing's syndrome

Insights Imaging (2024) Song L, Miao H, Zhu Zc, et al.

showed negative results on both ^{18}F-FDG PET/CT and ^{68}Ga-DOTATATE PET/CT (**A-F**). The fourth double-PET/CT-negative case in our study was shown in Figure E5. (**A-B**) Case 1, a 35-year-old woman. Chest CT showed a peripheral solid nodule (diameter 1.0cm) in the left upper lobe (arrows), with moderate enhancement of 53HU increments **(B)**, confirmed as typical carcinoid pathologically. (**C-D**) Case 2, a 35-year-old woman. Chest CT showed a peripheral solid nodule (diameter 0.7cm) in the left lower lobe (arrows), with obvious enhancement of 98HU increments **(D)**, confirmed as typical carcinoid pathologically. (**E-F**) Case 3, a 63-year-old man. Chest CT showed a peripheral solid nodule (diameter 0.6cm) in the right lower lobe (arrows), with obvious enhancement of 82HU increments **(F)**, confirmed as atypical carcinoid pathologically. (**G**) Scatter plot shows that double-PET/CT-negative tumors tend to be smaller in diameter on CT images.

Supplementary Reference

1. Arnaldi G, Angeli A, Atkinson AB, et al. Diagnosis and complications of Cushing's syndrome: a consensus statement. *The Journal of clinical endocrinology and metabolism*. Dec 2003;88(12):5593-602. doi:10.1210/jc.2003-030871

2. Zhu W, Cheng Y, Wang X, et al. Head-to-Head Comparison of (68)Ga-DOTA-JR11 and (68)Ga-DOTATATE PET/CT in Patients with Metastatic, Well-Differentiated Neuroendocrine Tumors: A Prospective Study. *Journal of nuclear medicine : official publication, Society of Nuclear Medicine*. Jun 2020;61(6):897-903. doi:10.2967/jnumed.119.235093

3. Decristoforo C, Melendez-Alafort L, Sosabowski JK, Mather SJ. 99mTc-HYNIC-[Tyr3]-octreotide for imaging somatostatin-receptor-positive tumors: preclinical evaluation and comparison with 111In-octreotide. *Journal of nuclear medicine : official publication, Society of Nuclear Medicine*. Jun 2000;41(6):1114-9.

4. Rindi G, Klimstra DS, Abedi-Ardekani B, et al. A common classification framework for neuroendocrine neoplasms: an International Agency for Research on Cancer (IARC) and World Health Organization (WHO) expert consensus proposal. *Modern pathology : an official journal of the United States and Canadian Academy of Pathology, Inc*. Dec 2018;31(12):1770-1786. doi:10.1038/s41379-018-0110-y
